# Acyloxyacyl hydrolase promotes the resolution of lipopolysaccharide-induced acute lung injury

**DOI:** 10.1371/journal.ppat.1006436

**Published:** 2017-06-16

**Authors:** Benkun Zou, Wei Jiang, Han Han, Jing Li, Weiying Mao, Zihui Tang, Qian Yang, Guojun Qian, Jing Qian, Wenjiao Zeng, Jie Gu, Tianqing Chu, Ning Zhu, Wenhong Zhang, Dapeng Yan, Rui He, Yiwei Chu, Mingfang Lu

**Affiliations:** 1 Department of Immunology, Key Laboratory of Medical Molecular Virology of Ministries of Education and Health, School of Basic Medical Sciences, and Shanghai Key Laboratory of Clinical Geriatric Medicine, Fudan University, Shanghai, China; 2 Shanghai Medical College, Fudan University, Shanghai, China; 3 Department of Pathology, School of Basic Medical Sciences, Fudan University, Shanghai, China; 4 Department of Thoracic Surgery, Zhongshan Hospital, Fudan University, Shanghai, China; 5 Department of Pulmonary Medicine, Shanghai Chest Hospital, Shanghai Jiao Tong University, Shanghai, China; 6 Departments of Infectious Diseases and Pulmonary Medicine, Huashan Hospital, Fudan University, Shanghai, China; DUMC, UNITED STATES

## Abstract

Pulmonary infection is the most common risk factor for acute lung injury (ALI). Innate immune responses induced by Microbe-Associated Molecular Pattern (MAMP) molecules are essential for lung defense but can lead to tissue injury. Little is known about how MAMP molecules are degraded in the lung or how MAMP degradation/inactivation helps prevent or ameliorate the harmful inflammation that produces ALI. Acyloxyacyl hydrolase (AOAH) is a host lipase that inactivates Gram-negative bacterial endotoxin (lipopolysaccharide, or LPS). We report here that alveolar macrophages increase AOAH expression upon exposure to LPS and that *Aoah*^*+/+*^ mice recover more rapidly than do *Aoah*^*-/-*^ mice from ALI induced by nasally instilled LPS or *Klebsiella pneumoniae*. *Aoah*^*-/-*^ mouse lungs had more prolonged leukocyte infiltration, greater pro- and anti-inflammatory cytokine expression, and longer-lasting alveolar barrier damage. We also describe evidence that the persistently bioactive LPS in *Aoah*^*-/-*^ alveoli can stimulate alveolar macrophages directly and epithelial cells indirectly to produce chemoattractants that recruit neutrophils to the lung and may prevent their clearance. Distinct from the prolonged tolerance observed in LPS-exposed *Aoah*^*-/-*^ peritoneal macrophages, alveolar macrophages that lacked AOAH maintained or increased their responses to bioactive LPS and sustained inflammation. Inactivation of LPS by AOAH is a previously unappreciated mechanism for promoting resolution of pulmonary inflammation/injury induced by Gram-negative bacterial infection.

## Author summary

Little is known about how MAMP molecules are degraded/inactivated and how inactivating them alters the course of inflammation *in vivo*. Our studies demonstrate that deficiency of acyloxyacyl hydrolase (AOAH, a host enzyme that inactivates LPS), prolongs pulmonary inflammation and acute lung injury in mice challenged with LPS or *Klebsiella pneumoniae*. This is the strongest evidence to date that disabling a MAMP molecule promotes recovery of infection-induced tissue injury. Degradation/inactivation of MAMP molecules is especially important because alveolar macrophages, unlike macrophages in many other tissues, do not become “tolerant” when they are exposed to LPS. In other words, they lack this general mechanism for down-regulating inflammation. Our findings indicate that inactivating LPS can compensate to a large extent for this deficiency–without AOAH, the LPS- or *Klebsiella*-challenged mice failed to resolve acute lung injury and were more likely to die. We reported previously that persistently bioactive LPS can maintain tolerance in peritoneal macrophages for many weeks in AOAH-deficient mice. The new findings show that the inability to inactivate a MAMP has different consequences in the lungs and peritoneum, in keeping with evidence that innate immune responses are compartmentalized. Inactivation of MAMP molecules can be an important and non-redundant prerequisite for inflammation resolution.

## Introduction

Acute lung injury (ALI) or its more severe form, acute respiratory distress syndrome, is a complex syndrome that may lead to acute respiratory failure and death. ALI has an average case-fatality rate of 38.5%; its incidence and mortality increase with age [1]. Although significant progress has been made toward understanding the pathophysiology of ALI, only supportive treatment, such as lung-protective ventilation, has reduced its mortality [2].

The most common risk factor for ALI is severe sepsis caused by bacterial or viral pneumonia [1]. During acute pulmonary infection, MAMPs or DAMPs (Danger-Associated Molecular Pattern) are detected by pattern recognition receptors (PRRs) on/in lung parenchymal and immune cells, eliciting innate immune responses that eliminate the invading microorganisms [3–6]. Uncontrolled inflammation and excessive accumulation/activation of leukocytes may lead to ALI and lung dysfunction.

Neutrophil accumulation is a cardinal feature of both acute inflammation and ALI [7]. MAMPS stimulate alveolar macrophages and airway epithelial cells to produce chemokines [8] that recruit neutrophils to the lung. In mice, KC (Keratinocyte chemoattractant, CXCL1), MIP-2 (macrophage inflammatory protein-2, CXCL2/3), the IL-23-IL17 axis and CXCL5 play this important role [9,10]. Neutrophils take up and kill bacteria, release proteases, produce reactive oxygen species and form neutrophil extracellular traps. Although these are critical actions for innate immune defense, excessive or prolonged accumulation of neutrophils may cause alveolar barrier damage and pulmonary edema, impairing gas exchange [2,9,11]. Sustained high neutrophil counts or IL-8 (functional homologue of mouse KC) levels in BALF have correlated with the severity of ALI while clearance of neutrophils from lung is a marker of ALI resolution and predicts a good outcome [9].

Since long-lasting inflammatory responses can lead to severe lung injury, timely resolution of the inflammation is critical for the host to minimize tissue damage and regain homeostasis [12–14]. Resolution of lung inflammation is an active and coordinated process that is believed to start with the degradation/inactivation of the inflammatory stimulus [15]. Perhaps surprisingly, how MAMPs are degraded/inactivated in the lung and how MAMP clearance affects the severity or duration of ALI have not been reported. Here we have studied the resolution of ALI induced by *Klebsiella pneumoniae*, an opportunistic Gram-negative pathogen that can cause severe pulmonary infection and ALI [16]. Much of the inflammatory response to these bacteria is induced by their lipopolysaccharides (LPS or endotoxin), potent MAMPs that are detected mainly by the MD-2-TLR4 receptor complex [17]. LPS, often used as a surrogate for infectious exposure, induces experimental ALI when it is introduced into the airway [18].

We found previously that LPS can be degraded and inactivated by a host enzyme, acyloxyacyl hydrolase (AOAH) [19,20]. AOAH removes the secondary fatty acyl chains from the lipid A moiety of LPS; the partially deacylated LPS becomes inactive or even may act as a LPS antagonist [21]. Our previous studies have shown that AOAH limits LPS-induced innate antibody production [22,23], prevents hepatosplenomegaly [24,25], and shortens endotoxin tolerance [26–28]; overexpression of AOAH protects mice from *E*. *coli* infection [29]. We hypothesized that AOAH, by degrading LPS in the lung, would also promote the resolution of ALI induced by LPS or Gram-negative bacteria.

We found that AOAH expression was highly inducible in alveolar macrophages after both *in vitro* and *in vivo* stimulation with LPS and other TLR agonists. AOAH promoted the resolution of ALI induced by LPS or *Klebsiella*, probably mainly by diminishing neutrophil chemoattractant production. Our results point to an important role for host inactivation of the bacterial cell wall lipopolysaccharide (LPS) in the timely resolution of pulmonary inflammation/injury induced by many Gram-negative bacteria.

## Results

### AOAH expression in the lung is dynamically boosted by LPS stimulation *in vitro* and *in vivo*

**Fig 1:**
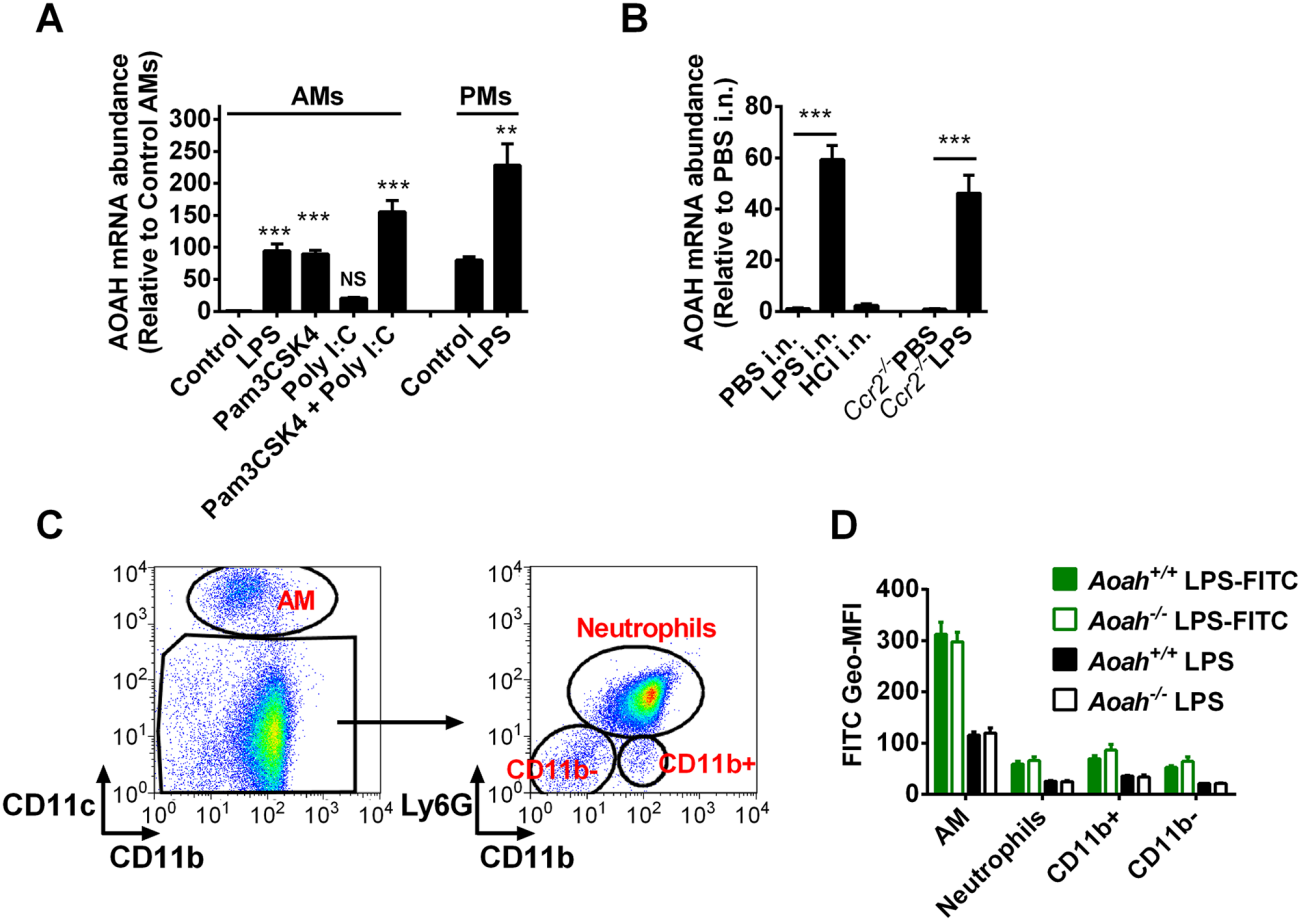
AOAH expression in alveolar macrophages is up-regulated upon LPS exposure *in vitro* and *in vivo*. (A) AMs and PMs were purified by letting the alveolar or peritoneal cells adhere to tissue culture plates, then they were cultured untreated (controls) or treated with 10 ng/ml LPS, 1 μg/ml Pam3CSK4, 10 μg/ml Poly I:C or 1 μg/ml Pam3CSK4 + 10 μg/ml Poly I:C for 18 hrs. AOAH mRNA was measured by using quantitative real-time PCR. The expression levels of control AMs were set to 1 and the relative expression levels of AOAH in treated AMs, control and treated PMs were calculated. Each group of treated AMs was compared with control AMs using one-way ANOVA. n = 8–10. AOAH expression in LPS-treated PMs was compared with that in untreated control PMs using Student’s t test. n = 6. (B) Mice were instilled i.n. with 50 μl PBS, 200 μg LPS in 50 μl PBS or 50 μl 0.2M HCl, AMs were isolated 18 hours later and purified, and AOAH mRNA was measured. LPS induced AOAH mRNA expression whereas HCl did not. The AOAH expression levels of AMs from PBS i.n. mice were set to 1. Data were combined from 3 experiments. One-way ANOVA test was used. n = 6–8. AOAH expression in AMs from PBS or LPS instilled *Ccr2*^*-/-*^ mice were compared using Student’s t test. n = 7–8. (C) Mice were instilled i.n. with LPS-FITC or LPS as a control and BAL was performed to obtain alveolar cells 18 hours later. The cells were stained and subjected to FACS. Gating strategy: Alveolar cells were divided into 4 groups according to their surface expression of CD11c, Ly6G and CD11b: CD11c^+^CD11b^lo^ AMs, CD11b^+^Ly6G^+^ neutrophils, CD11b^+^Ly6G^-^ mono-macrophages, and CD11b^-^Ly6G^-^ lymphocytes. (D) The geometric mean florescence intensity of FITC (Geo MFI) was measured in cells from mice instilled with LPS-FITC (Green bars) or LPS (Black bars). AMs have high autofluorescence. n = 6.

AOAH is expressed by several phagocytic cell types [20]. To test whether alveolar macrophages (AMs) also make AOAH, we obtained AMs from bronchoalveolar lavage fluid (BALF). Naïve AMs expressed much lower levels of AOAH mRNA than did naïve peritoneal macrophages (PM), yet LPS stimulation *in vitro* increased AM AOAH mRNA by over 90-fold (Fig 1A). TLR1/2 agonist Pam3CSK4 also enhanced AOAH mRNA expression, and the combination of Pam3CSK4 and TLR3 agonist Poly I:C was additive (Fig 1A). In contrast, naïve PMs expressed relatively high levels of AOAH and LPS slightly augmented AOAH expression (Fig 1A). When we introduced LPS intranasally (i.n.), AOAH expression in AMs increased by about 60-fold (Fig 1B). Although intranasally administered hydrochloric acid (HCl) can also induce lung inflammation, AOAH expression was not significantly increased (Fig 1B), suggesting that AOAH expression in AMs may be specifically induced by LPS and certain other MAMPs. The enhanced AOAH expression in AMs was not due to LPS-induced monocyte recruitment to the alveolar spaces, as we found that LPS induced AOAH expression to a similar extent in wild type mice and in CCR2-deficient mice (Fig 1B), which had diminished blood Ly6C^+^ monocytes [30] and recruited fewer CD11b^hi^ monocytes to the lung post LPS i.n. [31] (S1 Fig).

To identify the cells that take up LPS after i.n. instillation, we used LPS conjugated with FITC. Unlike macrophages from other sites, AMs express high levels of CD11c but low levels of CD11b and they have high autofluorescence [32]. Eighteen hours after instillation, LPS-FITC was mainly associated with CD11c^+^CD11b^lo^ autofluorescence^hi^ AMs, while CD11b^+^Ly6G^+^ neutrophils, CD11b^+^Ly6G^-^ mono-macrophages and CD11b^-^ Ly6G^-^ lymphocytes bound low amounts of LPS-FITC (Fig 1C and 1D). When we used trypan blue to quench extracellular FITC fluorescence, the FITC geometric florescence intensity was largely sustained, suggesting that the LPS-FITC had been internalized. Thus, after i.n. instillation, AMs took up more LPS than did the other cells in alveoli. *Aoah*^*+/+*^ and *Aoah*^*-/-*^ AMs took up comparable amounts of LPS (Fig 1D).

### AOAH ameliorates LPS-induced lung injury and promotes recovery

**Fig 2:**
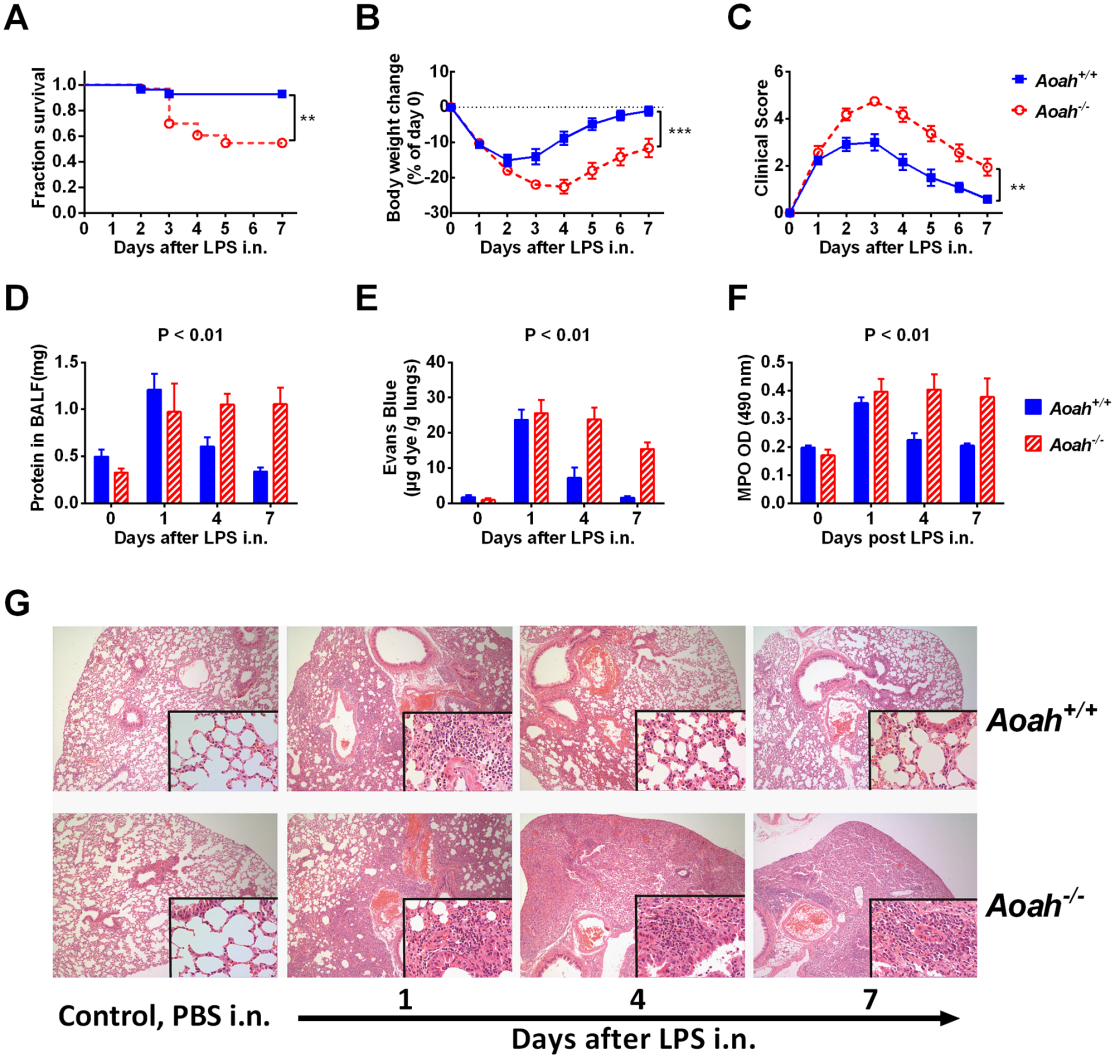
AOAH ameliorates LPS-induced lung injury and promotes recovery. (A) *Aoah*^*-/-*^ and *Aoah*^*+/+*^ mice were instilled with 200 μg LPS i.n. and their survival was monitored. Log-rank test was used. n = 28 (*Aoah*^*+/+*^) and 33 (*Aoah*^*-/-*^). (B) Body weight was measured daily in the mice that survived LPS instillation. Two-way ANOVA test was used. n = 26 (*Aoah*^*+/+*^) and 18 (*Aoah*^*-/-*^). (C) The mice that survived were also quantitatively assessed for signs of illness (weight loss, ruffled fur, ocular discharge, rapid shallow breathing, and lethargy). Data were combined from 5 experiments. Two-way ANOVA test was used. n = 26 (*Aoah*^*+/+*^) and 18 (*Aoah*^*-/-*^). (D) On days 0, 1, 4, and 7 after 150 μg LPS i.n., *Aoah*^*+/+*^ and *Aoah*^*-/-*^ mouse BALF was collected and the protein concentration was determined. Two-way ANOVA test was used. n = 6–10. (E) In separate experiments, mice were injected with Evans Blue 1 hr before euthanasia. The extravascular dye in the lungs was extracted and measured. Two-way ANOVA test was used. n = 3–6. (F) Before and 1, 4, and 7 days after 150 μg LPS i.n., MPO activity in lung homogenates was measured. Two-way ANOVA test was used. n = 8–9. (G) Hematoxylin-Eosin stained sections of paraformaldehyde-fixed lungs are shown. *Aoah*^*+/+*^ and *Aoah*^*-/-*^ mice were treated with PBS or 150 μg LPS, i.n. One, four and seven days later, their lungs were removed, fixed, sectioned and stained. The images represent at least 75% of whole sections. Original magnification X 40; insets, X 400. n = 3–4.

Using the LPS-induced acute lung injury (ALI) model, we studied whether AOAH influences ALI mortality and morbidity. To induce significant morbidity and mortality, we used a high dose of LPS (200 μg). After LPS instillation i.n., *Aoah*^*-/-*^ mice had greater mortality than did *Aoah*^*+/+*^ mice (Fig 2A). The *Aoah*^*-/-*^ mice that survived had greater and more prolonged weight loss (Fig 2B) and more severe and persistent clinical signs of illness than did *Aoah*^*+/+*^ survivors (Fig 2C), suggesting that AOAH-mediated LPS deactivation may play an important role in mitigating lung injury and promoting recovery.

An important feature of lung injury is the increased lung vascular and epithelial permeability that leads to accumulation of protein-rich fluid in alveolar spaces [2]. We studied whether *Aoah*^*-/-*^ mice had more alveolar barrier leakage than did *Aoah*^*+/+*^ mice after intranasal exposure to LPS. To induce significant yet non-lethal pulmonary injury, we instilled 150 μg LPS. One day after LPS i.n., both *Aoah*^*+/+*^ and *Aoah*^*-/-*^ mouse BALF had increased alveolar protein, suggesting the accumulation of extravascular protein in the alveolar space (Fig 2D). On days 4 and 7 post LPS, i.n., the protein concentrations in *Aoah*^*+/+*^ BALF had returned to normal levels, while *Aoah*^*-/-*^ BALF protein remained high, suggesting prolonged alveolar damage or delayed tissue repair (Fig 2D). We also measured alveolar barrier damage by injecting Evans blue i.v. and then measuring the extravascular dye in the lungs. (Fig 2E). *Aoah*^*-/-*^ mouse lungs had prolonged accumulation of Evans blue, suggesting prolonged alveolar leakage, in keeping with the results shown in Fig 2D. As neutrophil infiltration is known to be associated with alveolar damage [9], we measured myeloperoxidase (MPO) activity, a marker for neutrophil abundance, and found that *Aoah*^*-/-*^ mouse lungs had persistently high levels of MPO activity (Fig 2F). Thus, after LPS instillation i.n., *Aoah*^*-/-*^ mice had prolonged lung injury and delayed neutrophil clearance.

We compared the histological evidence of inflammation and injury in the lungs of *Aoah*^*+/+*^ and *Aoah*^*-/-*^ mice that had been exposed to intranasal LPS. *Aoah*^*+/+*^ and *Aoah*^*-/-*^ mice that had received PBS i.n. had thin alveolar walls with few neutrophils. One day after LPS i.n., peri-bronchial and peri-vascular leukocyte infiltrates and alveolar edema were observed in the lungs of both *Aoah*^*+/+*^ and *Aoah*^*-/-*^ mice (Fig 2G). The lung tissues close to bronchi had more inflammation and edema than did distal tissues (Fig 2G). On day 4 and 7, most leukocytes and edema were cleared in *Aoah*^*+/+*^ lungs, while *Aoah*^*-/-*^ lungs had persistent leukocyte infiltrates and severe edema (Fig 2G). Thus, AOAH promoted clearance of LPS-induced leukocyte accumulation and alveolar repair in the lung.

### AOAH hastens the resolution of LPS-induced inflammation in the lung

**Fig 3:**
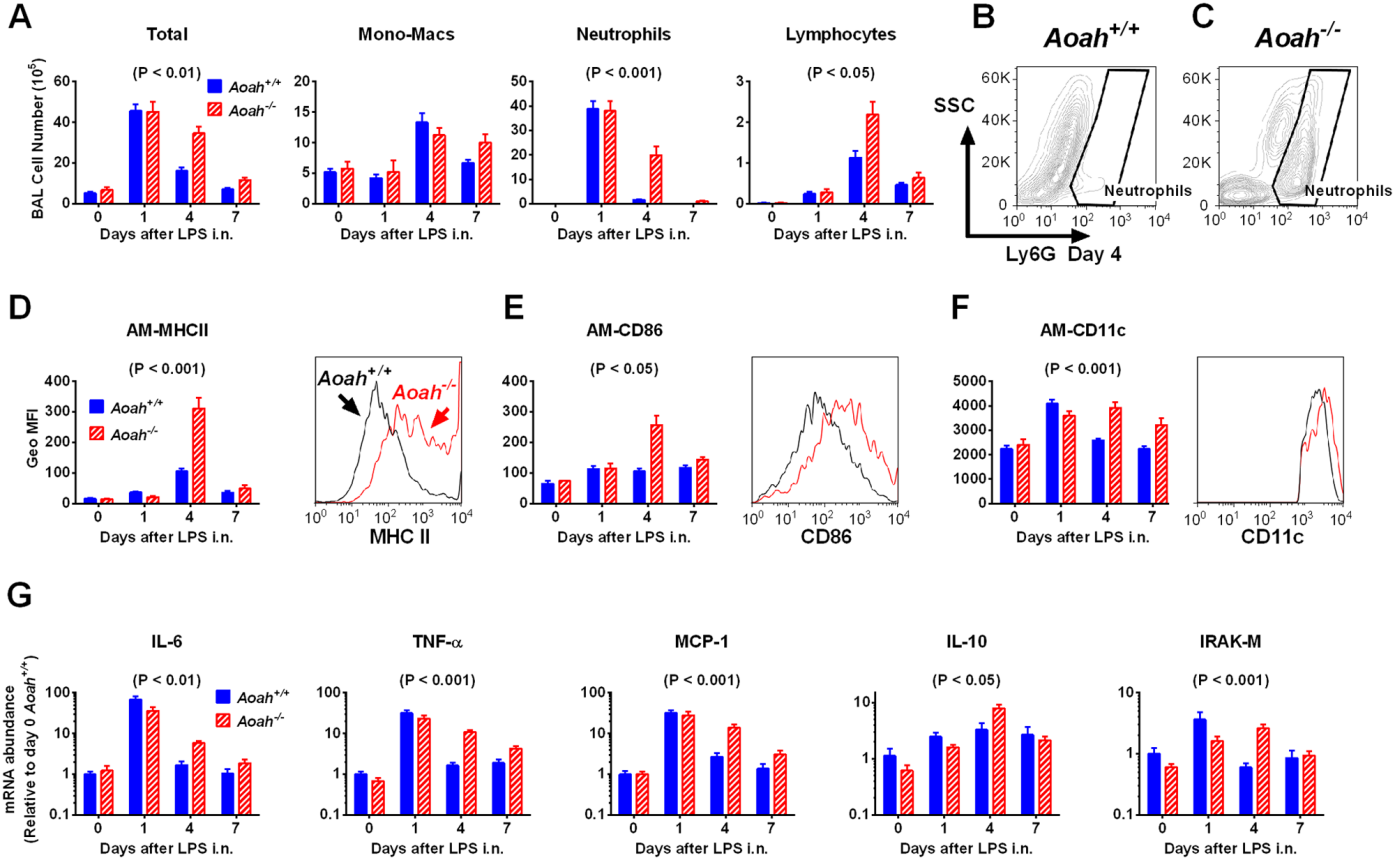
Intranasal LPS induces prolonged lung inflammation in *Aoah*^*-/-*^ mice. (A) *Aoah*^*+/+*^ and *Aoah*^*-/-*^ mice were untreated or treated with 10 μg LPS i.n. and 1, 4, and 7 days later their BALF was harvested. Total cell numbers were counted. The differential analysis of cells was performed by using cytospin and Wright-Giemsa staining. *Aoah*^*-/-*^ mice have prolonged immune cell infiltration in their airspaces in response to LPS i.n. Two-way ANOVA test was used. n = 6–11. (B and C) Representative flow cytometric plots of neutrophils (Ly6G+) in BALF from *Aoah*^*+/+*^ (B) and *Aoah*^*-/-*^ (C) mice 4 days after LPS i.n. (D) Geo MFI of MHC II on AMs from *Aoah*^*+/+*^ and *Aoah*^*-/-*^ (Left panel). Histogram overlay of MHCII expression of *Aoah*^*+/+*^ and *Aoah*^*-/-*^ AM (right panel). (E) CD86 expression on AMs. (F) CD11c expression on AMs. Combined data from 3 experiments (D-E). Two-way ANOVA test was used. n = 6–11. (G) *Aoah*^*+/+*^ or *Aoah*^*-/-*^ mice were treated with 10 μg LPS i.n. Their lungs were perfused and lavaged. Total RNA was extracted from lung homogenates, reverse transcribed, and mRNA abundance was measured by using quantitative real-time PCR. The mRNA levels in *Aoah*^*+/+*^ lungs (PBS, i.n., day 0) were set to 1, and the relative expression of other groups was calculated. Data were combined from 3 experiments. Two-way ANOVA test was used. n = 6–11.

To further study the impact that AOAH has on the resolution of lung inflammation, we treated mice with a low dose of LPS (10 μg) to induce significant inflammation without producing severe ALI. Almost all of the cells (over 90%) in BALF from PBS treated mice were alveolar macrophages (Fig 3A, day 0). One day after LPS i.n., the total cell numbers dramatically increased in both strains of mice, mainly due to neutrophil recruitment to the alveolar spaces (Fig 3A). Neutrophils in *Aoah*^*+/+*^ alveoli were largely cleared on day 4 (Fig 3A and 3B) and diminished on day 7 (Fig 3A), while in *Aoah*^*-/-*^ mice, alveolar neutrophils persisted on day 4 (Fig 3A and 3C), indicating that inflammation resolved more slowly in *Aoah*^*-/-*^ airspaces. We also observed increased numbers of lymphocytes in *Aoah*^*-/-*^ BALF on day 4; the monocyte-macrophage numbers were not significantly different between the two strains of mice (Fig 3A). We measured the immune cells in the lungs and found that, four and seven days after LPS exposure, *Aoah*^*-/-*^ lungs had more neutrophils, AMs, DCs and lymphocytes than did *Aoah*^*+/+*^ lungs (S2 Fig), consistent with the results found in airspaces.

To determine the activation states of the AMs, we measured macrophage activation markers MHCII and CD86 on AMs. Four days after LPS i.n., *Aoah*^*-/-*^ AMs (CD11c^hi^, hi autoflorescence cells) had significantly greater expression of MHCII (Fig 3D) and CD86 (Fig 3E) on the cell surface, suggesting that *Aoah*^*-/-*^ AMs were highly activated. CD11c expression was also maintained at high levels in *Aoah*^*-/-*^ AMs (Fig 3F). Thus, when the LPS-inactivating enzyme was absent, alveolar inflammation persisted and AMs were greatly activated.

LPS i.n. instillation induces pro-inflammatory and anti-inflammatory gene expression in the lung. To test whether AOAH influences LPS-induced cytokine gene expression, we measured cytokine mRNA abundance in lung homogenates. On day 1 after LPS exposure, LPS induced pro- and anti-inflammatory gene expression in both *Aoah*^*+/+*^ and *Aoah*^*-/-*^ lungs. When cytokine expression in *Aoah*^*+/+*^ mouse lungs started to return to basal levels on day 4, *Aoah*^*-/-*^ mouse lungs had significantly elevated cytokine gene mRNA, suggesting that AOAH promotes the cessation of inflammatory cytokine expression (Fig 3G). IRAK-M, a negative regulator of TLR signaling, also remained at higher levels on day 4 in *Aoah*^*-/-*^ mouse lungs (Fig 3G). The persistent expression of IRAK-M and anti-inflammatory cytokine IL-10 also suggests the presence of unresolved inflammation and these anti-inflammatory molecules may counteract inflammation to protect lung tissues. Taken together, these results strongly suggest that AOAH accelerates inflammation resolution in the lung.

### AOAH does not ameliorate HCl-induced lung inflammation

**Fig 4:**
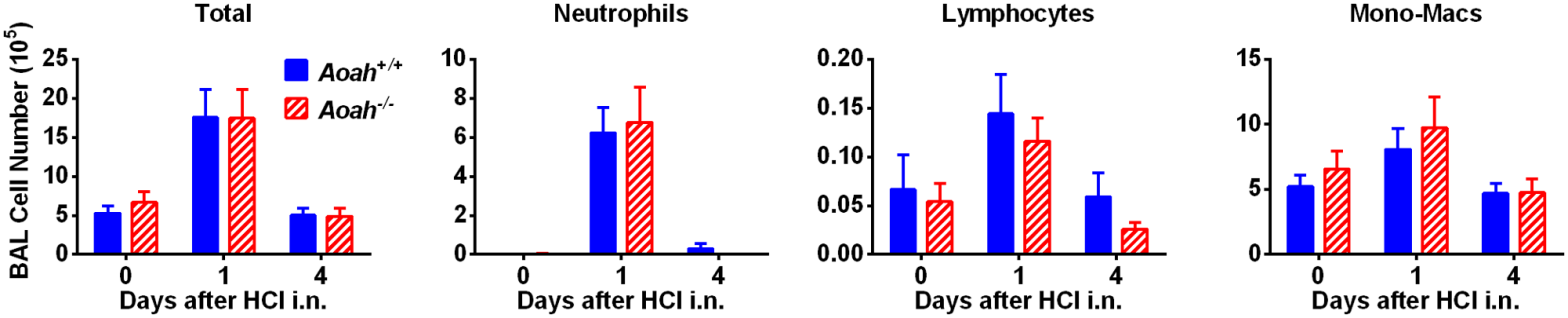
AOAH does not regulate HCl-induced lung inflammation. *Aoah*^*+/+*^ and *Aoah*^*-/-*^ mice were treated i.n. with 50 μl of 0.2 M HCl and followed for 4 days. Cells in BALF were counted and differentially analyzed. *Aoah*^*+/+*^and *Aoah*^*-/-*^ mice developed similar degrees of alveolar inflammation and the inflammation resolved at similar rates. Combined data from 3 experiments. Two-way ANOVA test was used. n = 9.

To test whether AOAH specifically ameliorates LPS-induced lung injury, we compared HCl-induced acute lung injury in *Aoah*^*+/+*^ and *Aoah*^*-/-*^ mice. HCl elicited similar leukocyte infiltration in the alveolar space and the inflammation resolved with comparable kinetics in *Aoah*^*+/+*^ and *Aoah*^*-/-*^ mice (Fig 4), suggesting that AOAH specifically modulates acute lung injury induced by its substrate, LPS.

### AOAH diminishes neutrophil chemoattractant expression

**Fig 5:**
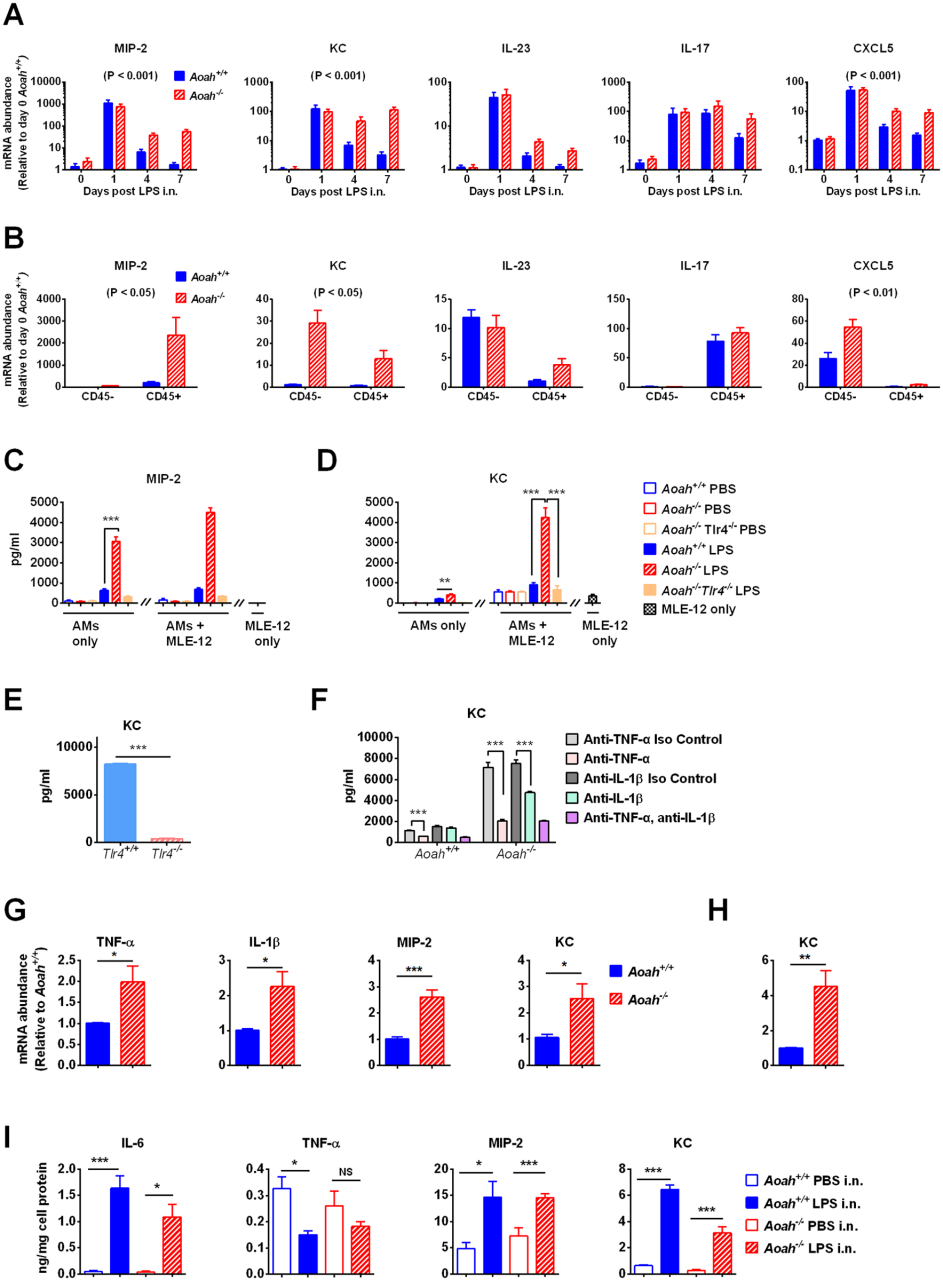
Persistent expression of neutrophil chemoattractants in *Aoah*^*-/-*^ lung. (A) Before and 1, 4, and 7 days after 150 μg LPS i.n., neutrophil chemoattractant MIP-2 (CXCL-2/3), KC (CXCL-1), IL-23, IL-17 and CXCL5 mRNA was determined in lung homogenates. Two-way ANOVA test was used. n = 8–9. (B) Seven days after 150 μg LPS i.n., CD45+ leukocytes and CD45- lung parenchymal cells were separated using MACS. MIP-2, KC, IL-23, IL-17 and CXCL5 mRNA expression was measured in both cell populations. Two-way ANOVA test was used. n = 4–5. (C, D) Seven days after PBS i.n. or 150 μg LPS i.n., AMs were isolated from *Aoah*^*+/+*^, *Aoah*^*-/-*^ and *Aoah*^*-/-*^*Tlr4*^*-/-*^ mice and either cultured alone or co-cultured with lung epithelial cell line MLE-12 for 6 hrs before MIP-2 and KC were measured in the media. One-way ANOVA test was used. n = 6. (E) Seven days after 150 μg LPS i.n., *Aoah*^*-/-*^*Tlr4*^*-/-*^ AMs were harvested and co-cultured with MLE-12. Naïve *Tlr4*^*+/+*^ or *Tlr4*^*-/-*^ AMs were added to the co-culture for 6 hrs. KC was measured in the culture media. Only *Tlr4*^*+/+*^ AMs promoted KC release. Student’s t test was used. n = 6. (F) Seven days after 150 μg LPS i.n., *Aoah*^*+/+*^ and *Aoah*^*-/-*^AMs were isolated and co-cultured with MLE-12 in the presence of anti-TNF-α, anti-IL-1β, both antibodies, or isotype control antibodies. KC in the culture media was measured by ELISA. Data were combined from 3 experiments. One-way ANOVA test was used. n = 8–11. (G) Seven days after 150 μg LPS i.n., AMs were isolated and RNA was extracted for qPCR. Student’s t test was used. n = 6–9. (H) Seven days after 150 μg LPS i.n., CD45- CD326+ lung epithelial cells were sorted using FACS and subjected to qPCR analysis. Student’s t test was used. n = 6–7. (I) Seven days after 150 μg LPS instillation, i.n., AMs were Isolated, allowed to adhere to plastic dishes and treated with 100 pg/ml LPS for 6 hrs before IL-6, TNF-α, MIP-2 and KC were measured in the culture media. Student’s t test was used to compare LPS-exposed AMs with control AMs of each strain of mice. n = 6.

Neutrophil infiltration is a sign of inflammation and neutrophil clearance is the most important marker of recovery from inflammation [9]. In response to intranasal LPS, alveolar neutrophil clearance was delayed in *Aoah*^*-/-*^ mouse lungs (Figs 2F and 3A–3C). We then studied the possible mechanisms for delayed neutrophil clearance, hypothesizing that the persistence of neutrophils in *Aoah*^*-/-*^ lung tissue is caused by increased recruitment, decreased apoptosis, or a combination of these factors. We tested neutrophil apoptosis 3 days after LPS i.n. using Annexin V and 7-AAD staining. Similar rates of neutrophil apoptosis were found in *Aoah*^*+/+*^ and *Aoah*^*-/-*^ alveoli (S3 Fig). We then compared the mRNA expression levels of neutrophil chemotactic chemokines/cytokines in *Aoah*^*+/+*^ and *Aoah*^*-/-*^ lungs. We instilled 150 μg LPS, which induced more prolonged neutrophil infiltration in *Aoah*^*-/-*^ lungs than did 10 μg LPS. Day 1 after LPS i.n., both strains of mice had dramatically elevated CXCL-2/3 (MIP-2), CXCL-1 (KC), IL-23, IL-17 and CXCL5 levels, while on day 4 and day 7, *Aoah*^*-/-*^ lungs had significantly higher CXCL-2/3 (MIP-2), CXCL-1 (KC) and CXCL5 expression than did *Aoah*^*+/+*^ lungs (Fig 5A), suggesting that persistent chemokine production sustained neutrophil recruitment and prevented neutrophil clearance.

To identify a source of these chemokines/cytokines, on day 7 after 150 μg LPS instillation i.n. we separated lung parenchymal cells (CD45-) from immune cells (CD45+) using MACS. CD45+ cells produced more MIP-2 and IL-17 than did CD45- cells; CD45- cells expressed more KC, IL-23 and CXCL5 than did CD45+ cells (Fig 5B). The expression levels of MIP-2, KC and CXCL5 were higher in *Aoah*^*-/-*^ cells than in their *Aoah*^*+/+*^ counterparts (Fig 5B). As we had found that AMs took up LPS (Fig 1C and 1D) and that AMs up-regulated AOAH expression upon exposure to LPS (Fig 1A and 1B), we surmised that AMs deacylate the LPS they ingest. We then hypothesized that when AOAH is missing, AMs cannot deacylate/inactivate the LPS that they take up. Bioactive LPS could then be released [27] to stimulate AMs to produce MIP-2 or to induce AMs and lung epithelial cells to secrete KC. We instilled PBS or 150 μg LPS in PBS i.n. to *Aoah*^*+/+*^ and *Aoah*^*-/-*^ mice and cultured their AMs *ex vivo* 7 days later. Explanted *Aoah*^*-/-*^ AMs, which had been exposed to LPS i.n., secreted significantly more MIP-2 than did *Aoah*^*+/+*^ AMs (Fig 5C). Co-culture with MLE-12, a commonly used murine lung epithelial line that preserves some features of alveolar epithelial cells [33], only slightly increased MIP-2 production (Fig 5C), consistent with the finding that CD45+ immune cells are the major source of MIP-2 (Fig 5B). Single culture of explanted LPS-exposed *Aoah*^*+/+*^ or *Aoah*^*-/-*^ AMs did not produce much KC in the media, while co-culture of AMs with MLE-12 dramatically enhanced KC secretion (Fig 5D); in the co-culture, *Aoah*^*-/-*^ AMs induced significantly higher levels of KC secretion than did *Aoah*^*+/+*^ AMs (Fig 5D), suggesting that AOAH-dependent inactivation of LPS dampens LPS-induced KC production by lung epithelial cells. To find out whether *Aoah*^*-/-*^ AM-derived LPS contributed to the enhanced MIP-2 and KC production, we treated the culture with a LPS inhibitor, polymyxin B, and found that polymyxin B largely reduced chemokine production (S4 Fig). When transwells were used to separate AMs and MLE-12, KC production was diminished, suggesting that cell-cell contact or proximity is important (S4 Fig).

Notably, AMs from LPS i.n. instilled *Aoah*^*-/-*^*Tlr4*^*-/-*^ mice did not induce KC secretion when co-cultured with MLE-12 (Fig 5D), whereas adding naïve *Tlr4*^*+/+*^ AMs but not *Tlr4*^*-/-*^ AMs to the co-culture of day 7 *Aoah*^*-/-*^*Tlr4*^*-/-*^ AMs and MLE-12 dramatically enhanced KC production (Fig 5E). These results strongly suggest that stimulation of AMs via TLR4 is required for KC production by lung epithelial cells. This experiment (Fig 5E) also confirmed that stimulatory LPS can be released from *Aoah*^*-/-*^*Tlr4*^*-/-*^ AMs. Since LPS stimulates AMs to produce pro-inflammatory cytokines, such as TNF-α and IL-1β, the cytokines may act on epithelial cells to secret chemokines [34]. As we failed to detect TNF-α and IL-1β in the cell culture using ELISA, which has detection limits of 7.8 and 15.6 pg/ml respectively, we tested the effects of anti-TNF-α and anti-IL-1β blocking antibodies in the co-culture. Blocking either TNF-α and IL-1β significantly reduced KC production; the TNF-α antibody had a more profound effect (Fig 5F). These results suggest that AM-derived pro-inflammatory cytokines stimulate epithelial cells to produce KC. In line with the co-culture results, we found that 7 days after LPS instillation i.n., *Aoah*^*-/-*^ AMs had significantly higher levels of TNF-α, IL-1β, MIP-2 and KC mRNA than did *Aoah*^*+/+*^ AMs (Fig 5G). FACS-sorted *Aoah*^*-/-*^ lung epithelial cells also had higher KC mRNA expression than did *Aoah*^*+/+*^ epithelial cells (Fig 5H). It has been shown that, distinct from macrophages form other sites, AMs that have been exposed to LPS *in vivo* are resistant to endotoxin tolerance or even increase their innate responsiveness [35–37]. To confirm that AMs maintain their responsiveness, 7 days after PBS or LPS instillation i.n., we explanted AMs and stimulated them with a low dose (100 pg/ml) of LPS. LPS-exposed AMs had increased IL-6, MIP-2 and KC responses and sustained or slightly decreased TNF responses (Fig 5I). Taken together, the data suggest that AMs can release the LPS they have taken up; when AOAH is lacking, the released bioactive LPS may directly act on AMs to secret MIP-2 and KC, and indirectly stimulate epithelial cells to produce KC. After exposure to LPS *in vivo*, instead of becoming tolerant, AMs maintained or increased their responsiveness to prolonged stimulation with low doses of LPS. The persistent chemokine/cytokine production may delay neutrophil clearance in *Aoah*^*-/-*^ mouse lungs.

### AOAH limits inflammation induced by Gram-negative bacteria

**Fig 6:**
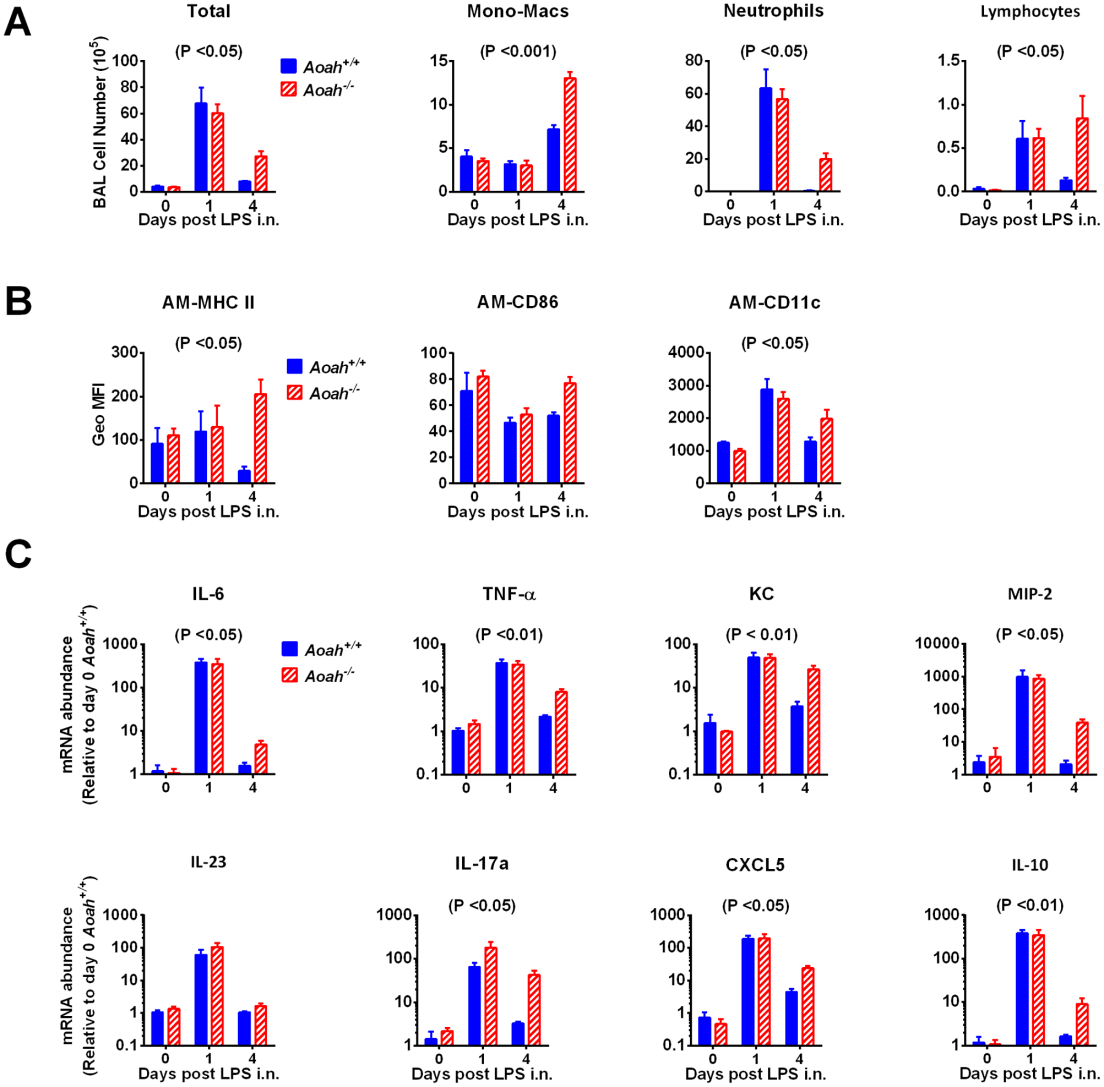
AOAH promotes the resolution of pulmonary inflammation induced by Gram-negative bacteria. (A) *Aoah*^*+/+*^ and *Aoah*^*-/-*^ mice were instilled i.n. with heat-inactivated 5 X 10^6^ *Klebsiella pneumoniae*. BALF was harvested for immune cell analysis before instillation and one and four days afterward. (B) The AM surface markers were measured using flow cytometry. (C) The lungs were excised and homogenized for cytokine and chemokine mRNA measurement. Data were combined from 2 experiments. Two-way ANOVA was used. n = 4–7.

We have shown that AOAH is required for the recovery from LPS-induced pulmonary inflammation/injury. We then asked whether AOAH was also important for recovery from pulmonary inflammation induced by Gram-negative bacteria, which contain LPS and many other TLR agonists [38]. We tested *Klebsiella pneumoniae*, a clinically important Gram-negative bacterium that may cause ARDS. As 1500 CFU of the *Klebsiella pneumoniae* strain we used killed 80% of *Aoah*^*+/+*^ mice, to induce significant pulmonary inflammation while preventing bacteria-induced death we instilled 5 X 10^6^ heat-killed *Klebsiella pneumoniae*. One day after instilling *Klebsiella pneumoniae*, *Aoah*^*-/-*^ and *Aoah*^*+/+*^ mouse airspaces had similar neutrophil infiltration, similar activation of AMs and their lungs had comparable cytokine and chemokine expression (Fig 6A–6C), while 4 days after instillation, in contrast, we found significantly more neutrophils, lymphocytes and macrophages in *Aoah*^*-/-*^ airspaces than in *Aoah*^*+/+*^ airspaces (Fig 6A). There was also higher expression of MHC II and CD11c on *Aoah*^*-/-*^ AMs than on *Aoah*^*+/+*^ AMs (Fig 6B) and *Aoah*^*-/-*^ lungs had greater IL-6, TNF-α, KC, MIP-2, IL-17a, CXCL5 and IL-10 mRNA expression than did *Aoah*^*+/+*^ lungs (Fig 6C). AOAH thus promoted recovery from lung inflammation induced by an opportunistic Gram-negative bacterial pathogen, indicating that even though many other TLR agonists were present, the detoxification of LPS by AOAH was required for resolution of inflammation.

### AOAH limits inflammation induced by chronic exposure to LPS

**Fig 7:**
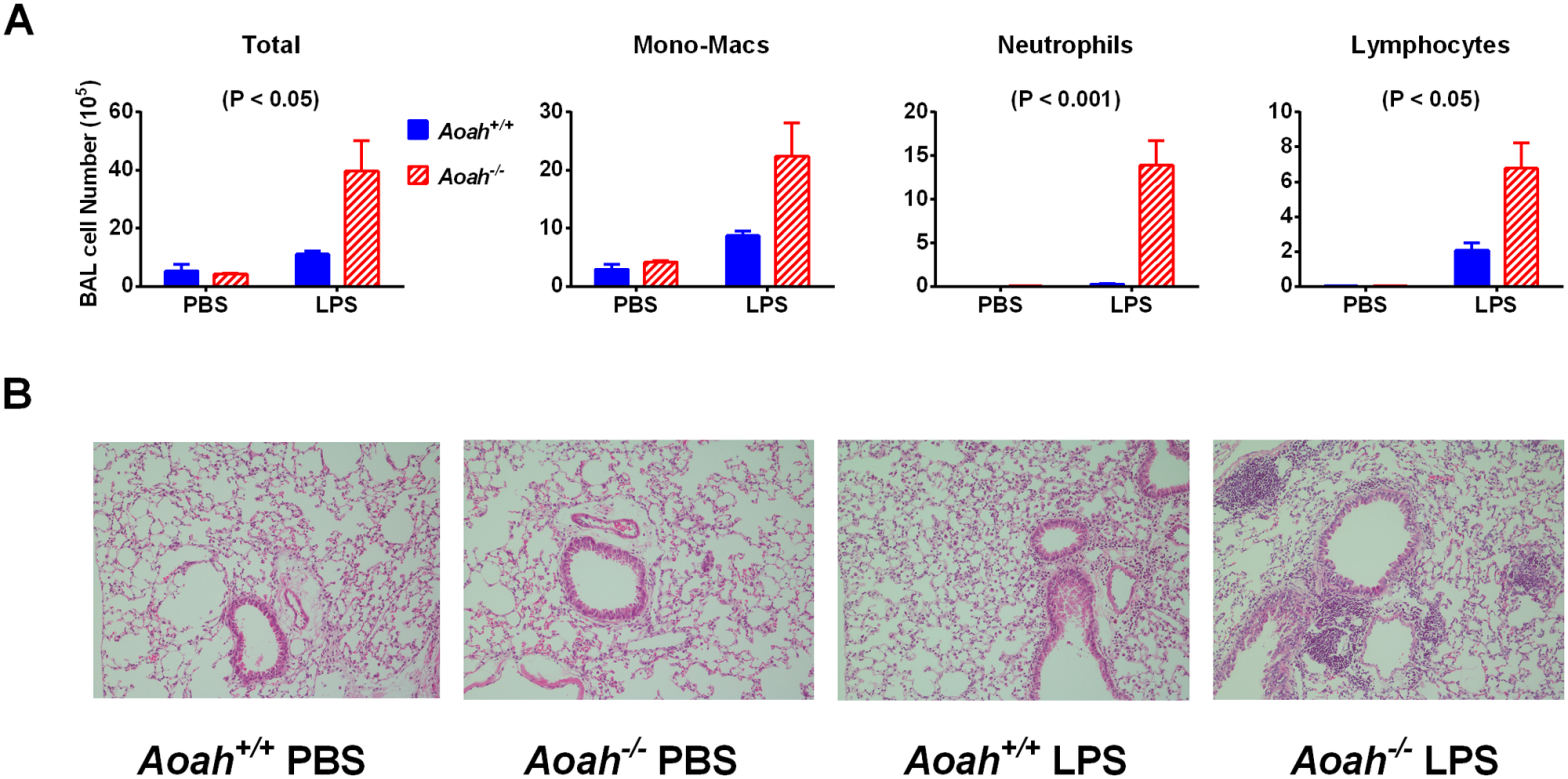
Chronic exposure to LPS leads to greater lung inflammation in *Aoah*^*-/-*^ lungs. Mice were treated with 10 μg LPS i.n. twice a week for 8 weeks. (A) *Aoah*^*-/-*^ mice had more inflammatory cells in their BALF than *Aoah*^*+/+*^ mice. Two-way ANOVA was used. n = 6–8. (B) AOAH deficiency aggravated lung inflammation after chronic exposure to LPS. The images represent at least 80% of the whole sections. n = 6–8. Magnification, 100 X.

To study whether chronic exposure to LPS may cause accumulative effects in the lungs of mice that cannot inactivate LPS, *Aoah*^*+/+*^ and *Aoah*^*-/-*^ mice were treated with 10 μg LPS i.n. every 3–4 days for 8 weeks. Four days after the last instillation, *Aoah*^*+/+*^ mice received LPS i.n. instillation had slightly more neutrophils and monocyte-macrophages than did control mice (PBS, i.n.), while *Aoah*^*-/-*^ mice had significantly more neutrophils, and lymphocytes in their BALF than did *Aoah*^*+/+*^ mice, demonstrating the exaggerated inflammation that occurs in *Aoah*^*-/-*^ mouse airspaces (Fig 7A). Pathological analysis also showed that *Aoah*^*-/-*^ lung tissue had more leukocyte infiltrates and aggregates (Fig 7B). Thus, AOAH controls chronic LPS exposure-induced lung inflammation.

## Discussion

**Fig 8:**
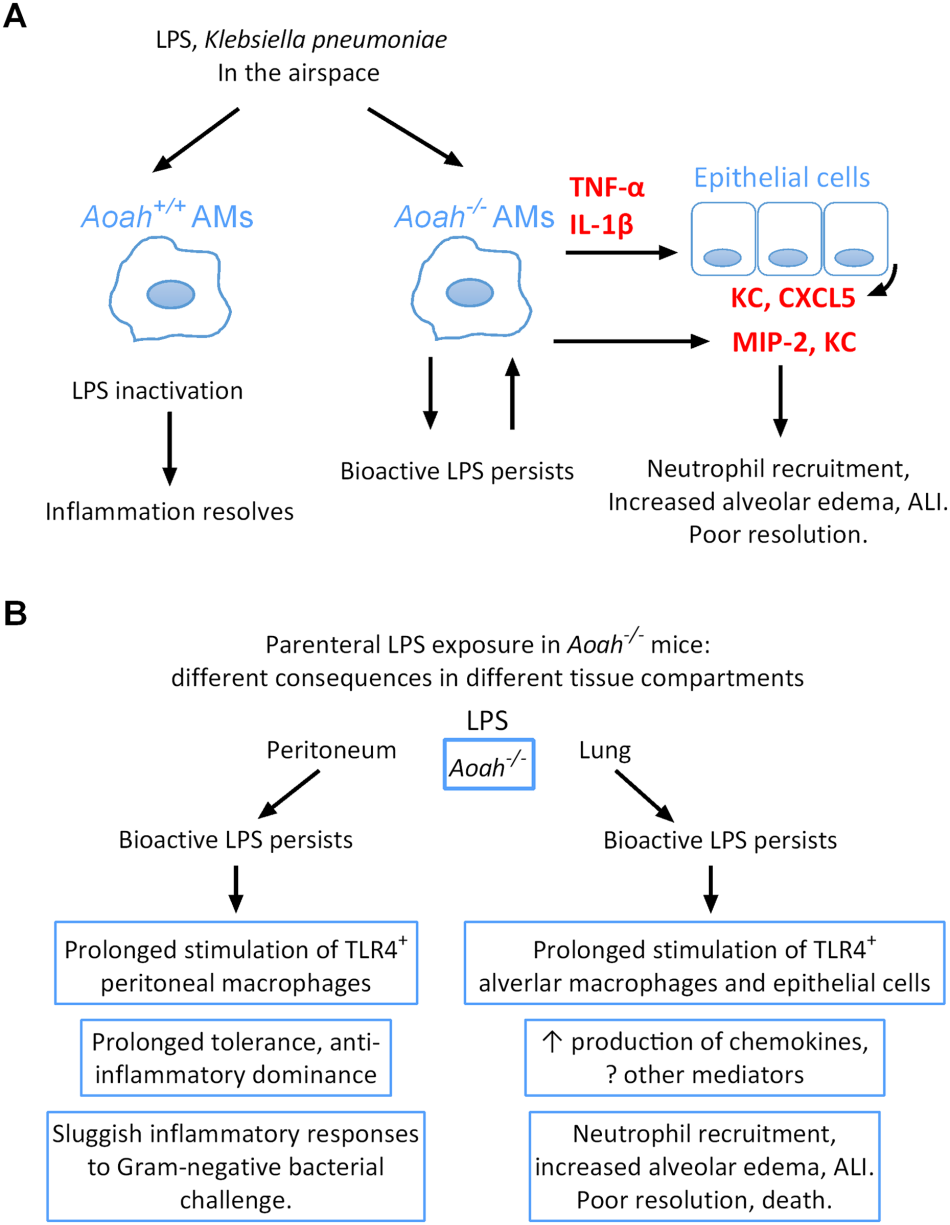
AOAH promotes the resolution of acute lung inflammation/injury induced by LPS or *Klebsiella pneumoniae*. (A) A working model. After LPS or *Klebsiella pneumoniae* are instilled i.n., AMs take up LPS or Gram-negative bacteria and degrade/inactive LPS. When AOAH is missing, AMs release bioactive LPS that can directly stimulate primed AMs to release cytokines and chemokines. Proinflammatory cytokines can further act on lung epithelial cells to produce more neutrophil chemoattactants, thus preventing neutrophil clearance and inflammation resolution. (B) AOAH deficiency has distinct consequences in different tissue compartments: prolonged macrophage tolerance in the peritoneum and persistent inflammation in the lung.

Recognizing and responding to MAMP molecules are critical steps in antimicrobial host defense. When the infection is under control, timely resolution of inflammation is important for the host to prevent severe tissue injury and regain homeostasis [15]. How MAMP molecules are degraded/inactivated in the lung and whether the clearance of MAMPs plays a role in the resolution of inflammation have been unclear. In this study, we investigated the roles that an endotoxin-degrading (deacylating) enzyme, AOAH, plays in the recovery from endotoxin-induce ALI. We found that AOAH expression in AMs was highly inducible in response to certain MAMP molecules (LPS and Pam3CSK4); that AMs took up LPS instilled i.n.; that AOAH shortened LPS-induced alveolar and lung inflammation, alveolar damage and ameliorated morbidity and mortality; and that, in mice that lack AOAH, bioactive LPS can be released from AMs and stimulate AMs as well as lung epithelial cells (indirectly) to produce neutrophil chemoattractants that may delay neutrophil clearance from the lung. AOAH also accelerated the resolution of inflammation induced by whole Gram-negative bacteria and diminished the inflammation induced by chronic LPS exposure. Thus, AOAH hastened the resolution of LPS or Gram-negative bacteria-induced lung inflammation/injury and promoted the restoration of homeostasis (Fig 8A). To our knowledge, this is the first evidence to date that disabling a MAMP molecule promotes recovery/resolution of infection-induced tissue injury.

Naïve mouse lungs do not express high levels of AOAH mRNA or AOAH enzymatic activity [39]. However, AOAH expression in AMs is highly inducible in response to LPS and other TLR agonists (such as Pam3CSK4) but not to HCl (Fig 1), consistent with the reported ability of TLR agonists to upregulate AOAH expression in dendritic cells [38]. These results indicate that when the lung detects MAMP molecules, not only are inflammatory responses elicited, but the program to destroy a key microbial signal molecule is also initiated. Indeed, this is the most dynamic up-regulation of AOAH expression observed so far [38,40,41].

When LPS was instilled i.n., AMs took up more LPS than did neutrophils and other cells in the airspace. Because AOAH expression in AMs is induced, LPS can be degraded inside AMs (Fig 1). Stamme and Wright have shown that AMs deacylate LPS and that surfactant protein A enhances their ability to do so [42]. Neutrophils, which also make AOAH and internalize LPS, may also play a role in deacylating instilled LPS [19,43]. Furthermore, AOAH may be secreted [44] and LPS can be degraded in extracellular fluids. Katz et al., showed that rabbit peritoneal exudate fluid was able to deacylate LPS [45] and Gioannini et al. reported that CD14 and LBP could present extracellular LPS for deacylation by AOAH [46]. Whether LPS can be degraded extracellularly in the lung requires further investigation.

We reported previously that LPS injected i.p. was taken up by peritoneal macrophages [26,27,47]. In *Aoah*^*-/-*^ mice, these macrophages released bioactive LPS into the peritoneal fluid and peritoneal macrophages remained tolerant for months [27]. Mice with prolonged tolerance mounted sluggish innate immune responses to Gram-negative bacterial challenge and died of uncontrolled infection [26]. In this study we found that *Aoah*^*-/-*^ AMs also took up LPS and released bioactive LPS. Distinct from peritoneal macrophages, however, upon exposure to LPS *in vivo*, AMs do not become tolerant [35,36]. Instead, they respond to repeated LPS exposure by maintaining or even increasing their responsiveness [37,48]; they are able to produce chemoattractants and cytokines in the presence of persistent LPS (Fig 5), thus delaying clearance of neutrophils and prolonging inflammation. For these cells (and presumably for other LPS-responsive cells that do not develop tolerance), MAMP inactivation is especially important to terminate inflammatory responses and prevent tissue injury. AOAH deficiency thus has distinct consequences in different body compartments: prolonged tolerance in the peritoneum yet sustained inflammation in the lung. As other inflammatory responses differ in the lung and peritoneum [35,49–52], so the impact of AOAH-mediated LPS inactivation is also compartmentalized (Fig 8B).

AOAH did not diminish the initiation of inflammatory responses to LPS in the lung, as demonstrated by the similar degrees of alveolar damage, MPO activity, lung pathology, BALF and lung inflammatory cell infiltration, and lung inflammatory cytokine or chemokine expression that occurred in *Aoah*^*+/+*^ and *Aoah*^*-/-*^ mice one day after LPS exposure (Figs 2, 3 and 5). The requirement to induce AOAH expression (Fig 1) and the slow kinetics of LPS-degradation by the enzyme *in vivo* [22–24] probably account for this observation. However, during the recovery phase, AOAH was important for inflammation resolution. Whereas *Aoah*^*+/+*^ mice started to recover from inflammation on day 4 when either high or low doses of LPS were instilled, *Aoah*^*-/-*^ mice demonstrated persistent inflammation and alveolar damage, delayed recovery and elevated morbidity and mortality (Figs 2, 3 and 5). Furthermore, after exposure to Gram-negative bacteria, *Klebsiella pneumoniae*, *Aoah*^*-/-*^ mouse lung also had prolonged inflammation (Fig 6). These results strongly suggest that LPS is one of the most potent bacterial MAMP molecules and that it must be inactivated to permit the resolution of Gram-negative bacteria-induced inflammation in the lung.

Neutrophils are among the first responders to infection. Clearance of neutrophils from tissue, as a marker of inflammation resolution, involves neutrophil apoptosis, efferocytosis by AMs, and cessation of further neutrophil recruitment [53]. Although studies have shown that LPS and inflammatory mediators inhibit neutrophil apoptosis and extend their functional longevity [54–56], in our ALI model we did not observe different neutrophil apoptotic rates in *Aoah*^*-/-*^ mouse lungs. Instead, we observed that the concentrations of neutrophil chemoattractants MIP-2, KC and CXCL5 failed to drop as quickly as they did in the lungs of *Aoah*^*+/+*^ mice (Figs 5 and 6). Using an *ex vivo* culture system, we found strong evidence that *Aoah*^*-/-*^ AMs could not inactivate the LPS they ingested and that they released LPS that could stimulate AMs and lung epithelial cells to produce neutrophil chemoattractants (Figs 5 and 6). AMs produce TNF-α and IL-1β upon LPS stimulation [34]. TNF-α is known to be able to induce epithelial cells to secret GM-CSF to promote tissue repair [57] and to enhance monocyte transmigration during influenza infection [58]. In this study, we found that AM-derived TNF-α and IL-1β stimulated KC production by epithelial cells, pointing to functional cross-talk between lung immune and parenchymal cells. In addition to macrophage-derived IL-1Ra and MMP12, which attenuate alveolar neutrophil recruitment [53], here we show that AMs promote neutrophil clearance by inactivating a MAMP molecule.

AOAH inactivates LPS by removing the secondary fatty acyl chains from the lipid A moiety, converting a hexaacyl (or hepta- or penta-acyl, in different LPSs) structure into one that has only four acyl chains. The tetraacyl moiety binds to MD-2 yet does not initiate signaling by TLR4; in fact, AOAH-treated LPS can be a potent LPS antagonist [21]. Although the lack of ALI observed in LPS-instilled *TLR4*^*-/-*^ mice argues strongly that signaling downstream of TLR4 is required, it is interesting to note that the recently reported cytosolic, caspase-based system for sensing LPS also recognizes hexaacyl lipid A; tetraacyl molecules bound to caspase 4/5/11 yet were poor agonists [59–61]. It is thus likely that AOAH-mediated deacylation/inactivation will also contribute to the resolution of inflammation induced by this mechanism.

In human studies, single nucleotide polymorphisms in the AOAH gene have been shown to be associated with asthma [62] and chronic rhinosinusitis in Chinese and Canadian Caucasian populations [63], suggesting that AOAH may regulate some pulmonary inflammatory responses in humans. It is important to note that another host enzyme, acidic mammalian chitinase (AMCase), also modulates lung inflammation. When AMCase was over-expressed in mouse lung, eosinophil infiltration after fungal challenge was diminished [64]. Moreover, a gain-of-function AMCase haplotype has been associated with asthma protection [65], while another study found that AMCase is causative in asthma [66]. These and our findings all point to the important roles that inactivating MAMP molecules may play in modulating MAMP-induced inflammation in the lung.

In conclusion, we report a previously unappreciated role of AOAH in promoting resolution of LPS- or Gram-negative bacteria-induced lung injury. In addition to the many other pro-resolution mechanisms, our study demonstrates that inactivating MAMP molecules can be critical for recovery from MAMP-induced lung injury. It would be interesting to investigate whether MAMP-inactivation is required for turning on the synthesis of “the specialized proresolving mediators” such as lipoxins and resolvins [12,13,67]. Interventions that accelerate the degradation of LPS and other MAMP molecules in tissue may promote resolution of inflammation.

## Materials and methods

### Mice

*Aoah*^*-/-*^, B6.B10ScN-Tlr4lps-del/JthJ (*Tlr4*^*−/−*^), *Aoah*^*-/-*^*Tlr4*^*-/-*^ and control *Aoah*^*+/+*^ C57BL/6J mice were obtained from the National Institutes of Health, USA (R.S. Munford). *Aoah*^*-/-*^ mice were generated as previously described [22]. The *Aoah* gene mutation had been backcrossed to C57Bl/6J mice for at least 10 generations. *Tlr4*^*-/-*^ mice have a 7 kb deletion in the TLR4 gene; the mutation had been backcrossed to C57Bl/6J for at least 6 generations. *Aoah*^*-/-*^ *Tlr4*^*-/-*^ were obtained by crossing *Aoah*^*-/-*^ mice with *Tlr4*^*-/-*^ mice. *Ccr2*^*-/-*^ C57BL/6J mice were from Fudan University Shanghai Medical College (Rui He). Age- and gender- matched 6–10 weeks old mice were used. The experiments in Figs 3 and 5 were repeated with similar results using littermates produced by *Aoah* heterozygous breeders. All mice were housed in a specific pathogen-free facility in the department of laboratory animal science of Fudan University.

### Ethics statement

All mice were studied using protocols approved by the Institutional Animal Care and Use Committee (IACUC) of Fudan University (approved animal protocol number 20140226–093). All protocols adhered to the Guide for the Care and Use of Laboratory Animals.

### Isolation and culture of AMs

Mice were anesthetized i.p. with 0.5% pentobarbital sodium (50 μg/g body weight) and exsanguinated by cutting the inferior vena cava. Bronchoalveolar lavage (BAL) was performed by cannulating the trachea with a 20-gauge catheter that was firmly fixed with a suture. The lung was then infused with 1 ml PBS containing 5 mM EDTA and the lavage fluid (bronchoalveolar lavage fluid, BALF) was extracted. This procedure was repeated 5 times and the BALF was combined. Cells in BALF were collected by centrifugation and then re-suspended in complete RPMI medium containing 10% fetal bovine serum (Hyclone), 2 mM glutamine, 100 U/ml penicillin, and 0.1 mg/ml streptomycin (Life Technologies). After adherence at 37°C for 2 hours and washing, mouse AMs were then untreated or treated with 10 ng/ml LPS O111 (Sigma), 1 μg/ml Pam3CSK4 (Invivogen, TLR1/2 agonist), 10 μg/ml Poly I:C (Invivogen, TLR3 agonist) or 1 μg/ml Pam3CSK4 + 10 μg/ml Poly I:C for 18 hours before AOAH mRNA analysis. Both Pam3CSK4 and Poly I:C had endotoxin levels < 0.001 EU/μg. We used Pam3CSK4 or Poly I:C to stimulate *Tlr4*^*+/+*^ and *Tlr4*^*-/-*^ peritoneal macrophages and comparable amounts of IL-6 were measured in the culture medium, confirming low endotoxin contamination. In some experiments, *Aoah*^*+/+*^ mice were instilled i.n. with 200 μg LPS or 50 μl 0.2M hydrochloric acid (HCl); their BALF was harvested 18 hrs later and AMs were purified for AOAH expression analysis.

### Internalization of LPS by macrophages

Mice were anesthetized and instilled with 20 μg *E*. *coli* LPS-O111-FITC (Sigma) i.n. Eighteen hours later, cells in airspaces were collected in BALF and stained with anti-CD11c, anti-CD11b and Ly6G antibodies (BD) to identify AMs and other cell populations. Cells were then washed, fixed and permeablized with Cytofix/Cytoperm buffer (BD). Anti-FITC antibody conjugated with FITC (Clone LO-FLUO-1, Life technologies) was added to magnify the signal. Cells were analyzed by flow cytometry. Trypan blue, which quenches extracellular FITC, did not decrease FITC Geo mean fluorescence intensity, suggesting that most of the LPS-FITC had been internalized by the cells.

### Quantitative assessment of clinical signs in mice

Before and after mice received 200 μg *E*. *coli* LPS, i.n., they were weighed daily and quantitatively assessed for their morbidity in a blinded manner. The clinical scoring system includes weight loss (> 10%), piloerection, ocular exudate, rapid shallow breathing and lethargy. Each finding was assigned a score of 1.

### Lung inflammation/injury models

LPS-induced acute lung injury: *Aoah*^*+/+*^ and *Aoah*^*-/-*^ mice were anesthetized and instilled with 10 μg, 150 μg or 200 μg LPS O111 (Sigma) in 40–50 μl PBS or PBS only (control) i.n. Ten μl LPS or PBS was instilled each time and the instillation was repeated for 4 or 5 times with 1–2 minute intervals. On day 1, 4, 7 after LPS i.n., mice were euthanized. After lungs were perfused with PBS, BALF was obtained and then lungs were excised for assays.

Hydrochloric acid (HCl)-induced lung injury: This model mimics pneumonitis caused by aspiration of gastric contents (aspiration pneumonitis [6,68]). Mice were anesthetized and then were instilled with 50 μl 0.2M HCl or PBS (control) i.n. On day 1, 4 after LPS i.n., mice were euthanized and immune cells in BALF were analyzed.

LPS-induced chronic lung inflammation: Mice were anesthetized and instilled with 10 μg *E*. *coli* LPS or PBS i.n. twice a week for eight weeks. Four days after the last LPS administration, BAL was performed for immune cell analysis.

### Analysis of BAL fluid

After BAL was performed, the BALF was centrifuged. The cell-free supernatant was used to measure protein using a bicinchoninic acid (BCA) kit (Pierce). The cell pellet was re-suspended in PBS and total cell numbers were counted using Cellometer (Nexelcom). Cell differentials were counted after cytospin and Wright-Giemsa staining. Three hundred cells, including monocytes-macrophages, neutrophils and lymphocytes were counted for each sample.

### Alveolar barrier damage

We tested alveolar leakage by measuring extravascular Evans blue in the lung. Briefly, mice were injected with 0.5 mg Evans blue (Sigma) i.v. 60 min before euthanasia. Lungs were then perfused to remove intravascular dye. Lungs were excised and homogenized in PBS. One volume of lung homogenate was incubated with 2 volumes of formamide and incubated at 60°C for 18 hrs before centrifugation. The optical density of the supernatant was measured at 620 nm and 740 nm using a Tecan reader. The concentrations of Evans blue were corrected for the presence of heme pigments using the following formula: A_620_ (corrected) = A_620_ (raw)—(1.1927 X A_720_) + 0.0071 [69]. The extravasated Evan blue dye concentrations were then calculated against a standard curve.

### Real time-PCR

RNA from AMs or lungs was isolated using RNA isolation kit (Tiangen) and reversely transcribed (Tiangen). The primers used for RT-PCR were listed in S1 Table. Actin was used as the internal control and the relative gene expression was calculated by the ΔΔCt quantification method.

### Flow cytometry

Cells in BALF were collected by centrifugation, incubated with Fc blocking antibody (purified anti-mouse CD16/32, BioLegend) to prevent binding of nonspecific FcγRIII/II, and then incubated with detection antibodies for 1 hr on ice. After washing, the samples were analyzed by CyAn-ADP (Beckman Coulter) and data were processed using Flow Jo software (TreeStar, Inc). All antibodies used for flow cytometry were anti-mouse antigens. Anti-CD11b (Clone M1/70), anti-CD11c (Clone N418), anti-Siglec F (Clone E50-2440), anti-MHCII (Clone M5/114.15.2), anti-Ly6G (Clone 1A8), anti-CD45 (Clone 30-F11), anti-CD86 (Clone FL1) were from BD Biosciences. Anti-F4/80 (Clone BM8) antibody was from BioLegend.

### Lung digestion and single cell preparation

To measure immune cells in the lung, the lung was perfused and then the trachea was cannulated with a 20-gauge catheter. The lung was instilled with 1 ml digestion buffer, which contained RPMI 1640, 1 mg/ml collagenase IV (Sigma) and 5U/ml DNase I (Sigma). The lung was excised, cut into 1 mm^3^ pieces and incubated in a culture tube containing 2 ml digestion buffer at 37°C for 1 hr with shaking. The digested lung tissues were filtered through a 70 mm cell strainer. Red blood cells were then lysed by using ACK lysis buffer (eBioscience). After total cell numbers were counted, cells were stained with antibodies and subjected to flow cytometric analysis of cell type.

### Histology

After lungs had been excised and fixed in 4% paraformaldehyde, they were sectioned and stained with hematoxylin and eosin (H&E). The samples were examined for inflammatory cell infiltration, tissue damage and alveolar edema by using a Nikon E200 microscope.

### MACS (Magnetic-activated cell sorting) and FACS (Fluorescence Activated Cell Sorting)

To identify the source of neutrophil chemoattractants, lung single cell suspensions were made as described above. Cells were counted and separated into CD45+ or CD45- cells by using anti-CD45 antibody-conjugated magnetic beads (Miltenyi Biotec) according to the manufacturer’s instructions. In another experiment, CD45- CD326+ alveolar epithelial cells were sorted using FACS. The purity of CD45+, CD45- cells and CD45- CD326+ was above 95% by flow cytometric analysis.

### Myeloperoxidase (MPO)

Lung samples were homogenized in a buffer containing 0.5% hexadecyltrimethylammonium bromide (HTAB), 0.5 mM EDTA, 500 mM potassium phosphate buffer (pH 7.0). The lung tissue slurry was pelleted and the supernatant was added to MPO assay buffer, which contained 50 mM potassium phosphate buffer (pH 7.0), 0.0005% H_2_O_2_ (w/w), 0.168 mg/ml o-dianisidine dihydrochloride (ODH). After 25 mins incubation at 25°C, the plate was read at a wavelength of 490nm (Tecan).

### AM culture and co-culture with MLE-12

To find out whether AMs can release the LPS they ingested, BALF was collected from *Aoah*^*+/+*^ and *Aoah*^*-/-*^ mice seven days after they had received 150 μg LPS intranasally. The AMs were isolated and 0.5 X 10^6^ AMs were cultured or co-cultured with the same number of mouse lung epithelial cells (line MLE-12, ATCC CRL2110) for 6 hrs, then MIP-2 and KC concentrations in the culture media were measured by using ELISA (R&D). In some experiments LPS inhibitor polymyxin B (20 μg/ml) (Sigma), anti-mTNF-α (Clone MP6-XT22, BD) or anti-IL-1β (Clone, B122, Biolegend) blocking antibodies (10 μg/ml), or isotype control antibodies (10 μg/ml, BD and Biolegend respectively) was added to the media. In other experiments, AMs were separated from MLE-12 cells by transwells (Corning). We also instilled 150 μg LPS i.n. to *Aoah*^*-/-*^ *Tlr4*^*-/-*^ mice, and seven days later we co-cultured their AMs with MLE-12 cells plus naïve *Tlr4*^*+/+*^ or *Tlr4*^*-/-*^ AMs for 6 hrs. KC concentrations were measured in the culture media.

### Bacteria-induced pulmonary inflammation

*Aoah*^*+/+*^ and *Aoah*^*-/-*^ mice were instilled i.n. with heat-killed (boiled for 15 minutes) 5 X 10^6^ *Klebsiella pneumoniae subsp*. *pneumoniae* (ATCC, 43816, Serotype 2). Before instillation and 1 or 4 days after, their BALF were harvested for immune cell analysis. The inoculation dose was tested to be able to elicit significant pulmonary inflammation.

### Statistics

One-way ANOVA test was used to compare multiple groups. Unpaired Student’s t test (two-tailed) was used for comparisons between two groups with significance set at p < 0.05. To compare survival proportions, Log-rank test was used. To compare two trends over time, two-way ANOVA test was used. *, P < 0.05; **, P < 0.01; ***, P < 0.001. Data are presented as mean ± standard error of the mean (SEM).

## Supporting information

S1 Fig *Ccr2*^*-/-*^ mice had diminished monocyte recruitment to air spaces in response to intranasal LPS instillation. (A) Blood was collected from *Ccr2*^*+/+*^ and *Ccr2*^*-/-*^ mice. After red blood cells were lysed, cells were stained with CD11b and F4/80 and subjected to flow cytometric analysis. n = 3. (B) Mice were instilled with 10 μg LPS i.n. Four days later, their BALF was obtained and the cells were stained with CD11b and CD11c before FACS analysis. *Ccr2*^*-/-*^ mice had fewer circulating monocytes and recruited fewer monocytes (CD11b^hi^, CD11c^lo or hi^) [31] to the lung in response to intranasal LPS instillation. Student’s t test was used. n = 3.
(TIF)

S2 Fig *Aoah*^*-/-*^ mouse lungs had more sustained immune cell infiltration than did *Aoah*^*+/+*^ mouse lungs. *Aoah*^*+/+*^ or *Aoah*^*-/-*^ mice were treated with PBS or 10 μg LPS i.n for 1, 4, or 7 days. Their lungs were perfused, lavaged and digested to make single cell suspensions. Total cell numbers were counted. Cells were stained with various antibodies and subjected to flow cytometric analysis. (A) Gating strategy. Lymphocytes and innate lymphoid cells (ILCs), CD45^+^, SSC^lo^; AMs, CD45^+^, CD11c^hi^, SiglecF^+^; Neutrophils, CD11b^+^, Ly6G^+^; DCs, CD11c^hi^, MHCII^hi^; Monocytes-Macrophages, CD11c^lo^, MHCII^mid^. (B) The graphs show the number of leukocytes per lung. Two-way ANOVA test was used. n = 7–8.
(TIF)

S3 Fig *Aoah*^*+/+*^ and *Aoah*^*-/-*^ alveolar neutrophils had similar rates of apoptosis. Mice were instilled with 10 μg LPS, i.n. Three days later, their alveolar cells were harvested and neutrophil apoptosis was measured by using Annexin V and 7-AAD staining. Two-way ANOVA test was used. n = 7.
(TIF)

S4 Fig *Aoah*^*-/-*^ AM-derived LPS stimulates chemokine production. *Aoah*^*+/+*^, *Aoah*^*-/-*^ and *Aoah*^*-/-*^*Tlr4*^*-/-*^ mice were treated with 150 μg LPS i.n. Seven days later, their AMs were isolated and either cultured alone or co-cultured with the lung epithelial cell line MLE-12 for 6 hrs. In some experiments, LPS inhibitor polymyxin B (20 μg/ml) was added. In other experiments, AMs were separated from MLE-12 by transwells. (A) Secreted MIP-2 was measured in the culture media. (B) Secreted KC was measured. One-way ANOVA test was used. n = 3–6.
(TIF)

S1 Table Primers used for qPCR. (DOCX)
