# Supplementary figures and images for: Acyloxyacyl hydrolase promotes the resolution of lipopolysaccharide-induced acute lung injury
Source: PLoS Pathog. 2017 Jun 16;13(6):e1006436. doi: 10.1371/journal.ppat.1006436 (PMC5489216; doi:10.1371/journal.ppat.1006436)

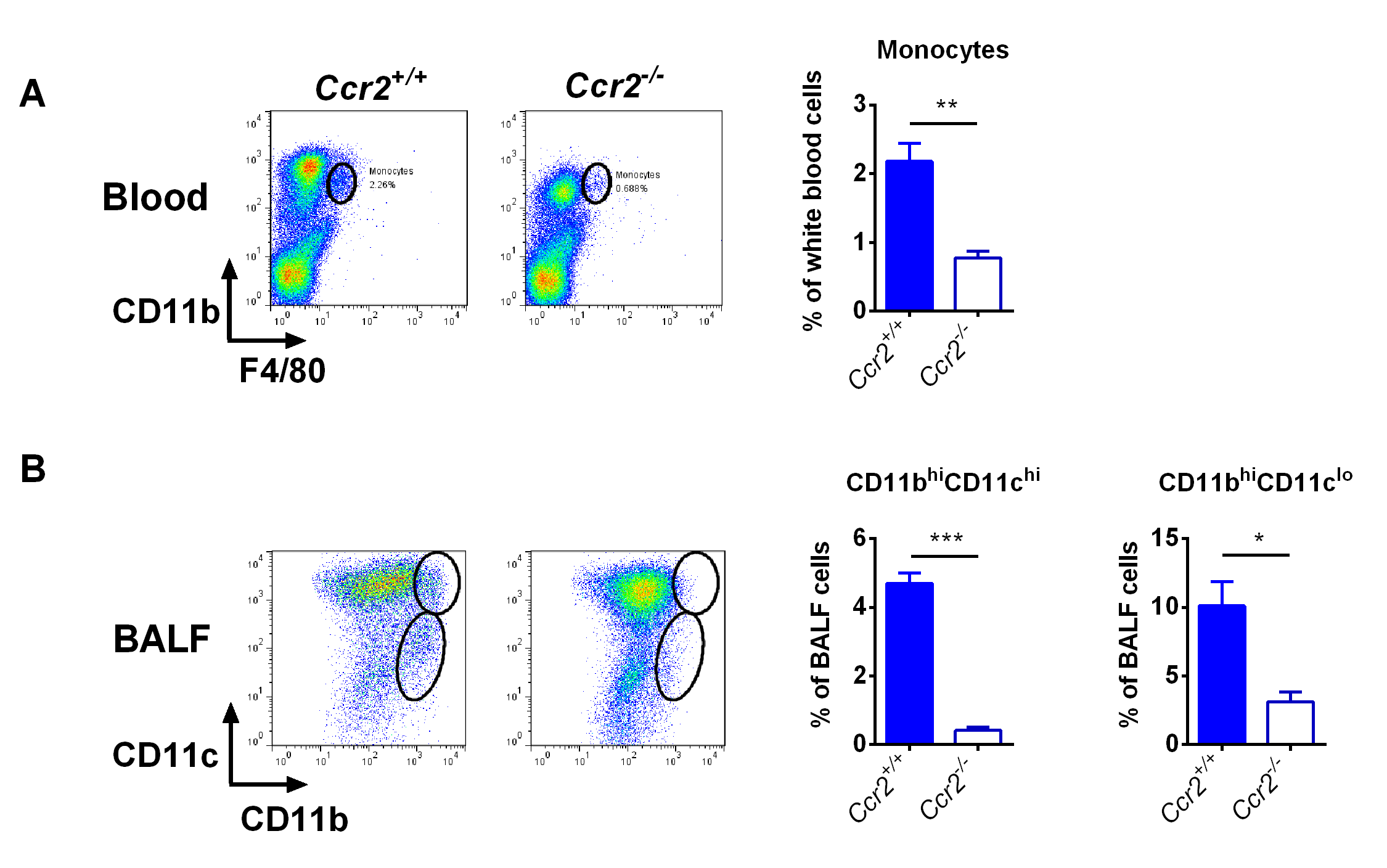

Supplement: S1 Fig — (A) Blood was collected from Ccr2+/+ and Ccr2-/- mice. After red blood cells were lysed, cells were stained with CD11b and F4/80 and subjected to flow cytometric analysis. n = 3. (B) Mice were instilled with 10 μg LPS i.n. Four days later, their BALF was obtained and the cells were stained with CD11b and CD11c before FACS analysis. Ccr2-/- mice had fewer circulating monocytes and recruited fewer monocytes (CD11bhi, CD11clo or hi) [31] to the lung in response to intranasal LPS instillation. Student’s t test was used. n = 3. (TIF) [file ppat.1006436.s001.tif]

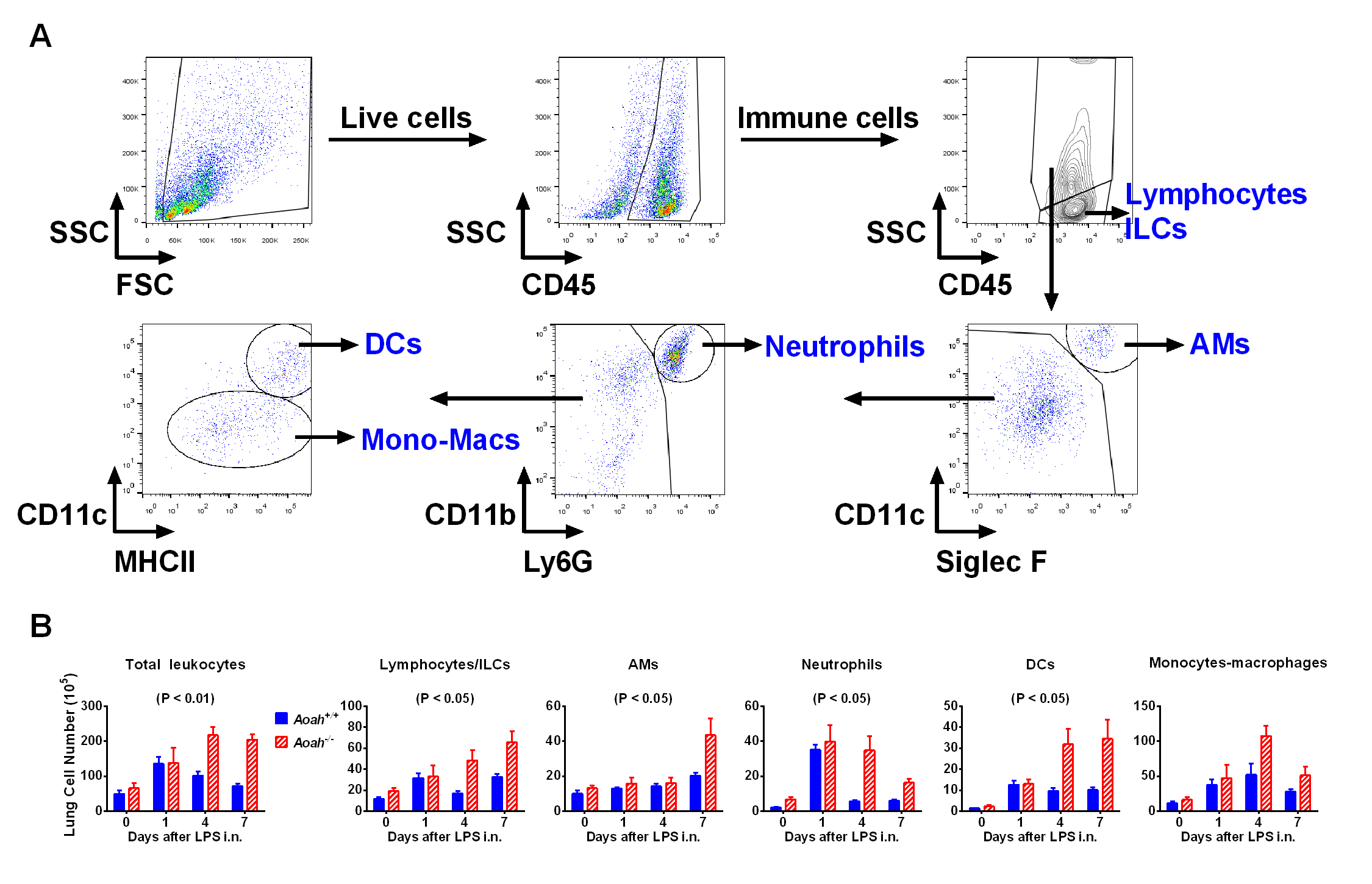

Supplement: S2 Fig — Aoah+/+ or Aoah-/- mice were treated with PBS or 10 μg LPS i.n for 1, 4, or 7 days. Their lungs were perfused, lavaged and digested to make single cell suspensions. Total cell numbers were counted. Cells were stained with various antibodies and subjected to flow cytometric analysis. (A) Gating strategy. Lymphocytes and innate lymphoid cells (ILCs), CD45+, SSClo; AMs, CD45+, CD11chi, SiglecF+; Neutrophils, CD11b+, Ly6G+; DCs, CD11chi, MHCIIhi; Monocytes-Macrophages, CD11clo, MHCIImid. (B) The graphs show the number of leukocytes per lung. Two-way ANOVA test was used. n = 7–8. (TIF) [file ppat.1006436.s002.tif]

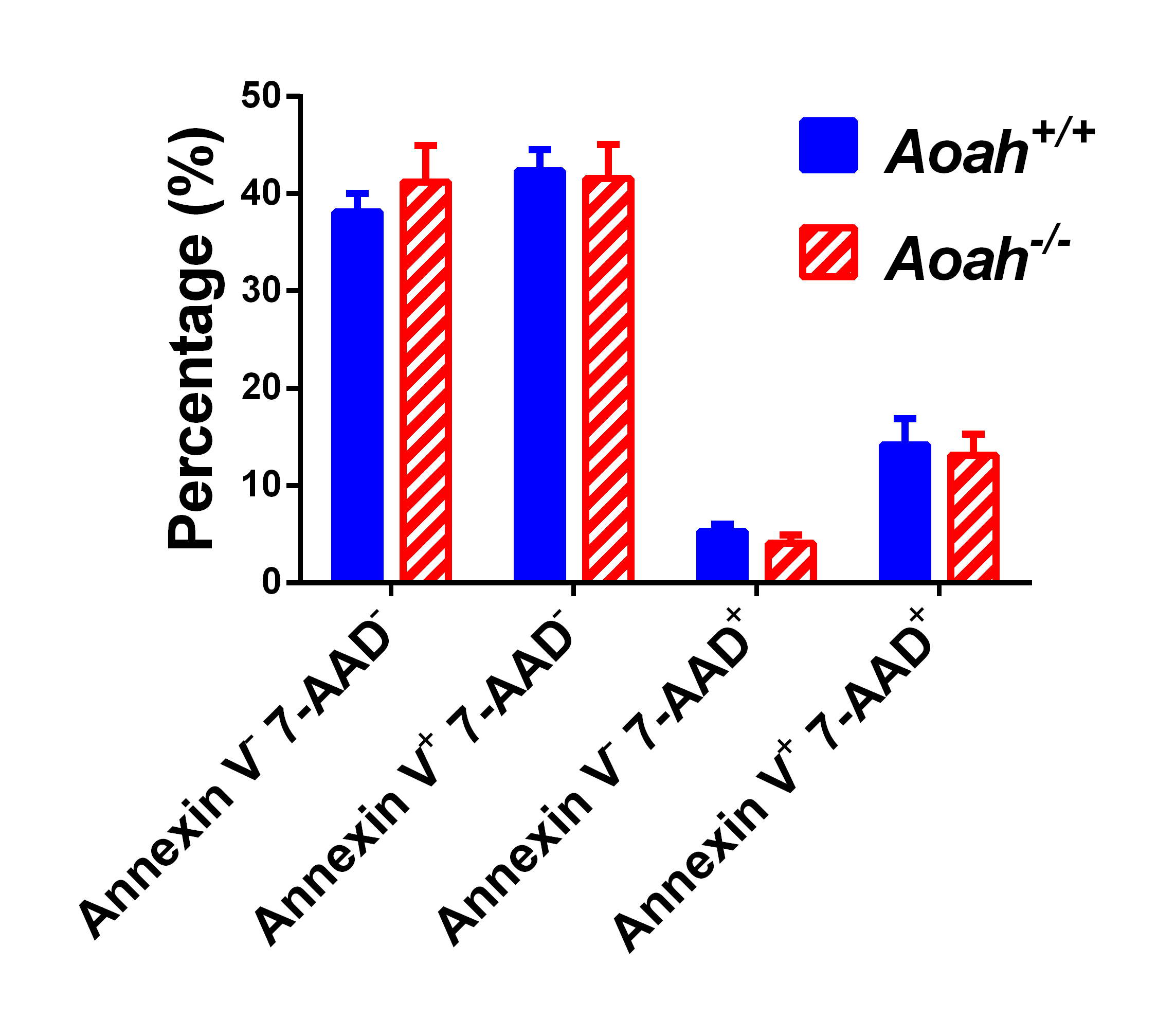

Supplement: S3 Fig — Mice were instilled with 10 μg LPS, i.n. Three days later, their alveolar cells were harvested and neutrophil apoptosis was measured by using Annexin V and 7-AAD staining. Two-way ANOVA test was used. n = 7. (TIF) [file ppat.1006436.s003.tif]

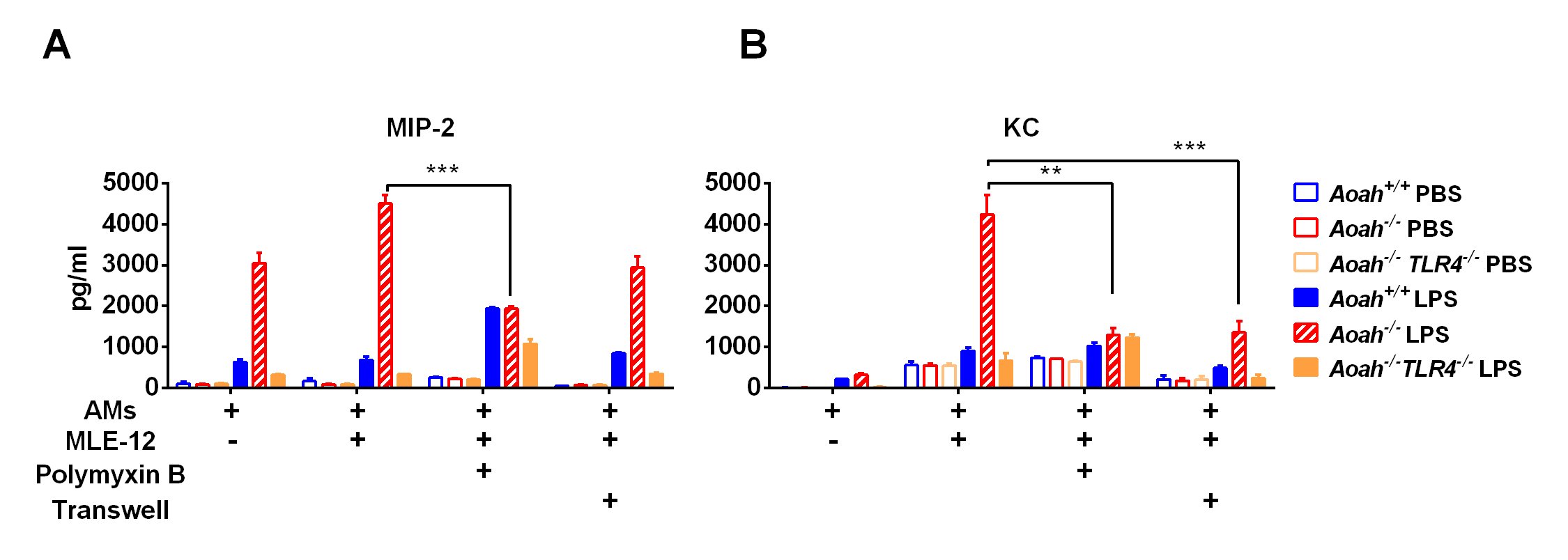

Supplement: S4 Fig — Aoah+/+, Aoah-/- and Aoah-/-Tlr4-/- mice were treated with 150 μg LPS i.n. Seven days later, their AMs were isolated and either cultured alone or co-cultured with the lung epithelial cell line MLE-12 for 6 hrs. In some experiments, LPS inhibitor polymyxin B (20 μg/ml) was added. In other experiments, AMs were separated from MLE-12 by transwells. (A) Secreted MIP-2 was measured in the culture media. (B) Secreted KC was measured. One-way ANOVA test was used. n = 3–6. (TIF) [file ppat.1006436.s004.tif]
