# Supplementary material for: Acyloxyacyl hydrolase promotes the resolution of lipopolysaccharide-induced acute lung injury
Source: PLoS Pathog. 2017 Jun 16;13(6):e1006436. doi: 10.1371/journal.ppat.1006436 (PMC5489216; doi:10.1371/journal.ppat.1006436)
Supplement: S1 Table — (DOCX) [file ppat.1006436.s005.docx]

**S1 Table.** Primers used for qPCR.

| Mouse gene symbols | Forward primer sequence | Reverse primer sequence |
| --- | --- | --- |
| Actin | 5’-GGCTGTATTCCCCTCCATCG-3’ | 5’-CCAGTTGGTAACAATGCCATGT-3’ |
| AOAH | 5’-GTTTTCCCAACGCTGCGGGG-3’ | 5’-TGGCCTTCTGCCCGGGTACA-3’ |
| IL-6 | 5’-ATCGTGGAAATGAGAAAAGAGTTGT-3’ | 5’-AAGTGCATCATCGTTGTTCATACA-3’ |
| TNF-α | 5’-CATCTTCTCAAAATTCGAGTGACAA-3’ | 5’- TCAGCCACTCCAGCTGCTC-3’ |
| MCP-1 | 5’-GGCTCAGCCAGATGCAGTTAA-3’ | 5’-CCTACTCATTGGGATCATCTTGCT-3’ |
| IL-10 | 5’-GCTGGACAACATACTGCTAACC-3’ | 5’-ATTTCCGATAAGGCTTGGCAA-3’ |
| IRAK-M | 5’-TCCCACCTGAGGTGAAGCAT-3’ | 5’-TGTGACATTGGCTGGTTCCA-3’ |
| MIP-2 (CXCL2/3) | 5’-AGCTACATCCCACCCACACAG-3’ | 5’-AAAGCCATCCGACTGCATCT-3’ |
| KC (CXCL1) | 5’-CAAGAACATCCAGAGCTTGAAGGT-3’ | 5’-GTGGCTATGACTTCGGTTTGG-3’ |
| CXCL5 | 5’-GCCCTACGGTGGAAGTCATA-3’ | 5’-GTGCATTCCGCTTAGCTTTC-3’ |
| IL-23 | 5’-CAGCAGCTCTCTCGGAATCTC-3’ | 5’-TGGATACGGGGCACATTATTTTT-3’ |
| IL-17 | 5’-TGTGAAGGTCAACCTCAAAGTC-3’ | 5’-AGGGATATCTATCAGGGTCTTCATT-3’ |
